# Conformational state-dependent regulation of GABA_A_ receptor diffusion and subsynaptic domains

**DOI:** 10.1016/j.isci.2022.105467

**Published:** 2022-10-29

**Authors:** Zaha Merlaud, Xavier Marques, Marion Russeau, Ursula Saade, Maelys Tostain, Imane Moutkine, Marc Gielen, Pierre-Jean Corringer, Sabine Lévi

**Affiliations:** 1 INSERM UMR-S 1270, Sorbonne Université, Institut du Fer à Moulin, 75005 Paris, France; 2 Institut Pasteur, Université Paris Cité, CNRS UMR 3571,Channel-Receptors Unit, Paris, France; 3 Sorbonne Université, 21, Rue de l’Ecole de Médecine, 75006 Paris, France

**Keywords:** Molecular biology, Neuroscience, Cellular neuroscience

## Abstract

The efficacy of GABAergic synapses relies on the number of postsynaptic GABA_A_ receptors (GABA_A_Rs), which is regulated by a diffusion capture mechanism. Here, we report that the conformational state of GABA_A_Rs influences their membrane dynamics. Indeed, pharmacological and mutational manipulations of receptor favoring active or desensitized states altered GABA_A_R diffusion leading to the disorganization of GABA_A_R subsynaptic domains and gephyrin scaffold, as detected by super-resolution microscopy. Active and desensitized receptors were confined to perisynaptic endocytic zones, and some of them were further internalized. We propose that following their activation or desensitization, synaptic receptors rapidly diffuse at the periphery of the synapse where they remain confined until they switch back to a resting state or are internalized. We speculate that this allows a renewal of activatable receptors at the synapse, contributing to maintain the efficacy of the synaptic transmission, in particular on sustained GABA transmission.

## Highlights

• Conformational changes impair GABA__A__R diffusion and SSD
• Conformational changes reduce GABA_A_R and gephyrin at synapses
• Active or desensitized receptors are exchanged with resting ones at the synapse

## Graphical abstract

**Figure:**
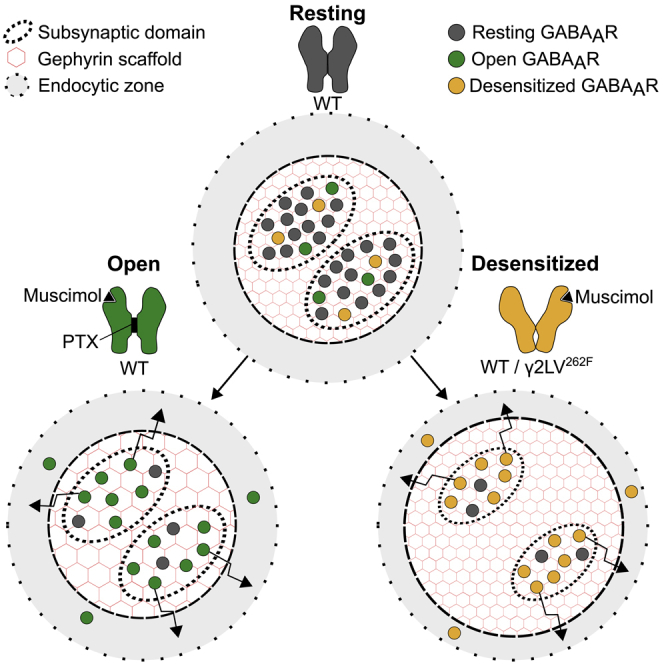

## Introduction

Type A GABA receptors (GABA_A_Rs) belong to the pentameric ligand-gated ion channel family and are the main inhibitory neurotransmitter receptors in the mammalian brain. The efficacy of inhibitory synaptic transmission relies in part on the number of GABA_A_Rs present in the postsynaptic membrane opposite to GABA-releasing presynaptic boutons. The number of GABA_A_Rs at synapses is rapidly regulated by a “diffusion-capture” mechanism in which receptors alternate between rapid diffusion into the extrasynaptic plasma membrane and slowing down and confinement to the synapses (Bannai et al., 2009; Choquet and Triller, 2013). Interaction of the GABA_A_R with its main scaffolding protein, gephyrin, is responsible for its confinement and synaptic aggregation (Jacob et al., 2005; Mukherjee et al., 2011; Petrini et al., 2014; Renner et al., 2012). Regulation of receptor lateral diffusion is considered the first mechanism for adapting the number of receptors at synapses in response to synaptic demand (Choquet and Triller, 2013; Triller and Choquet, 2008; Triller et al., 2020). The lateral diffusion of GABA_A_Rs is regulated by neuronal activity (Bannai et al., 2009,^2015^; Muir et al., 2010; Petrini and Barberis, 2014; Petrini et al., 2014), providing the molecular basis underlying synaptic plasticity at inhibitory GABAergic synapses of the hippocampus (Petrini and Barberis, 2014; Petrini et al., 2014). Activity is thought to control the diffusion and number of GABA_A_Rs at synapses by regulating, in particular, receptor binding to gephyrin through the modulation of receptor and/or gephyrin phosphorylation, with subsequent influence on the conformation of these proteins (Saliba et al., 2012). Of interest, the diffusion-capture of GABA_A_Rs can be regulated by changes in the allosteric conformation of the receptor independently of changes in neuronal activity. We and others reported that negative and positive benzodiazepine-like allosteric modulators of GABA_A_Rs decrease and increase the diffusion-capture of GABA_A_Rs, respectively (Gouzer et al., 2014; Lévi et al., 2015). It is well established that GABA_A_Rs alternate between three major conformations termed allosteric states, a resting state that is mainly in the absence of agonist, an active state with an open channel that is transiently populated on agonist application, and a desensitized state that is stabilized on prolonged exposure of the agonist (Gielen and Corringer, 2018). Because benzodiazepines act by displacing the allosteric equilibria, these results suggested that the diffusion of GABA_A_Rs are sensitive to benzodiazepine-induced conformational changes. A similar effect was demonstrated for AMPA-type glutamate receptors (Constals et al., 2015), with a rapid exchange of desensitized receptors with resting ones acting to regulate the amplitude of postsynaptic responses (Constals et al., 2015).

Here, to further investigate the impact of GABA_A_R conformation on its diffusion, we tested the receptor synaptic diffusion and subsynaptic domain (SSD) organization in the presence of agonists and antagonists. We demonstrate that favoring active or desensitized states alter the diffusion of GABA_A_R γ2-and α1-subunit containing receptors, leading to the disorganization of GABA_A_R SSDs and gephyrin scaffold, as well as receptor confinement in endocytic pits where some of them were internalized. Therefore, GABAergic synapses may rapidly exchange active and desensitized receptors with resting ones to maintain the fidelity of GABAergic neurotransmission. This mechanism may be particularly relevant for prolonged receptor activation leading to long-lasting desensitized states up to tens of seconds (Overstreet et al., 2000; Petrini et al., 2011).

## Results

### Promoting GABA_A_R activation or desensitization increases the diffusion of the γ2 subunit and reduces its synaptic confinement

We studied the GABA_A_R membrane dynamics using quantum dot-based single-particle tracking (QD-SPT) in primary cultures of hippocampal neurons. Since the lateral diffusion of the GABA_A_R is rapidly tuned by changes in glutamatergic transmission (Bannai et al., 2009,^2015^; Muir et al., 2010; Petrini and Barberis, 2014; Battaglia et al., 2018), the impact of GABA_A_R conformational states on its diffusion was explored in the presence of the Na^+^ channel blocker tetrodotoxin TTX (1 μM), the ionotropic glutamate receptor antagonist kynurenic acid (KYN, 1 mM), and the group I/group II mGluR antagonist R,S-MCPG (500 μM) to silence glutamatergic transmission. To favor specific allosteric states of GABA_A_Rs, ligands were added to the cultured neurons prior to perform QD-SPT experiments. Experiments were performed in three conditions: (1) In the absence of GABA_A_R ligand, in a condition where weak or no channel activity is observed, indicating that GABA_A_Rs are majoritary in the resting state. This condition is referred to as the “control” condition (see STAR Methods). (2) In the presence of a saturating concentration of the full agonist muscimol (100 μM, called muscimol condition), that is known to mainly stabilize the receptor in the desensitized state on prolonged exposure (Mortensen et al., 2010). (3) In the presence of both muscimol (100 μM) and picrotoxin (PTX, 500 μM), called muscimol + PTX condition (Gielen and Corringer, 2018). PTX is a well characterized channel blocker that was recently shown to prevent, at least partially, the desensitization of the receptor. Indeed, extensive structural analysis of GABA_A_R and their closely related glycine receptor (GlyR) show that desensitization is caused by a narrowing of the channel at its cytoplasmic end. PTX is known to bind at this level, and was shown to disfavor the desensitization conformational change by a “foot-in-the-door” mechanism as inferred from electrophysiological (Gielen et al., 2015) and structural data (Masiulis et al., 2019; Kumar et al., 2020). In the presence of both muscimol and PTX, the receptor thus mainly oscillates between the resting and the active states. This latter state, though, does not conduct ions through the plasma membrane because of the pore-blocking properties of PTX (Gielen and Corringer, 2018).

We first checked that the presence of TTX and glutamate receptor antagonists in our experiments minimize Ca^2+^ influx. Although NMDA or PTX application in the absence of TTX + KYN + MCPG increased [Ca^2+^]_i_ in hippocampal neurons (Figures S1A-S1E), muscimol + PTX or muscimol application in the presence of TTX + KYN + MCPG (named TKM condition) did not increase [Ca^2+^]_i_ in hippocampal neurons (Figures S1D-S1E). Therefore, we concluded that the conditions to promote the open and desensitized states of the GABA_A_R have no major effect on neuronal activity.

**Figure 1:**
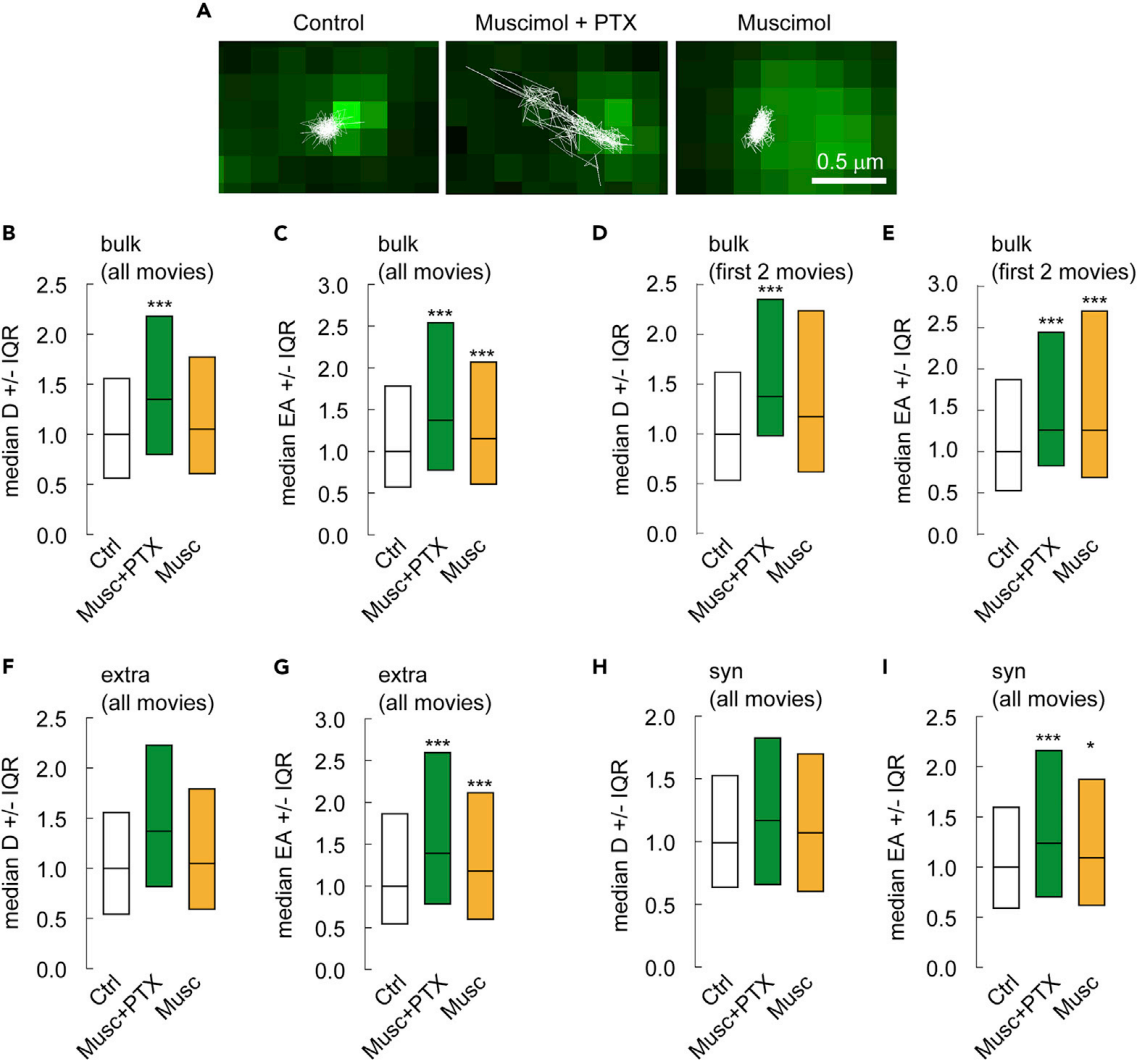
Promoting the active and desensitized conformation of the GABA_A_R influences the lateral diffusion of the γ2 subunit (A) Trajectories of the γ2 subunit of the GABA_A_R (white) overlaid with fluorescent clusters of Gephyrin-FingR-eGFP (green) to identify trajectories at extrasynaptic sites and at inhibitory synapses. Scale bar, 0.5 μm. These examples illustrate an increased exploration for receptors maintained in presence of muscimol + PTX (Musc + PTX) or muscimol (Musc) to promote the active and desensitized conformations, respectively. (B–C) Diffusion coefficients (B) and explored area (C) (for bulk population of QDs, from all movies) of GABA_A_Rγ2 in Musc + PTX (green) and Musc (orange) conditions. (B) Ctrl, n = 705 QDs, Musc + PTX, n = 630 QDs, p = 7.6 10^−12^, Musc, n = 728 QDs, p = 0.06, 3 cultures; (C) Ctrl, n = 2115 QDs, Musc + PTX, n = 1883 QDs, p = 5.1 10^−20^, Musc, n = 2184 QDs, p = 1.2 10^−5^, 3 cultures. (D–E) Diffusion coefficients (D) and explored area (E) (for bulk population of QDs, from the first two movies) of GABA_A_Rγ2 in Musc + PTX (green) and Musc (orange) conditions. (D) Ctrl, n = 187 QDs, Musc + PTX, n = 108 QDs, p = 4.4 10^−6^, Musc, n = 216 QDs, p = 0.0525, 3 cultures; (E) Ctrl, n = 561 QDs, Musc + PTX, n = 330 QDs, p = 7.4 10^−8^, Musc, n = 648 QDs, p = 6.6 10^−6^, 3 cultures. (F–G) Diffusion coefficients (F) and explored area (G) (for extrasynaptic population of QDs, from all movies) of GABA_A_Rγ2 in Musc + PTX (green) or Musc (DS, orange) conditions. (F) Ctrl, n = 546 QDs, Musc + PTX, n = 540 QDs, p = 4.2 10^−11^, Musc, n = 570 QDs, p = 0.2, 3 cultures; (G) Ctrl, n = 1634 QDs, Musc + PTX, n = 1616 QDs, p = 1.5 10^−18^, Musc, n = 1706 QDs, p = 9.1 10^−5^, 3 cultures. (H–I) Diffusion coefficients (H) and explored area (I) (for synaptic population of QDs, from all movies) of GABA_A_Rγ2 in Musc + PTX (green) or Musc (DS, orange) conditions. (H) Ctrl, n = 163 QDs, Musc + PTX, n = 94 QDs, p = 0.3, Musc, n = 162 QDs, p = 0.4, 3 cultures; (I) Ctrl, n = 485 QDs, Musc + PTX, n = 270 QDs, p = 2.2 10^−3^, Musc, n = 482 QDs, p = 2.5 10^−2^, 3 cultures. In B-I, data are presented as median values ± 25–75% IQR. In all graphs, values were normalized to the corresponding control values. ∗, p < 5.0 10^−2^; ∗∗∗, p < 1.0 10^−3^ (Kolmogorov-Smirnov test). See also Figure S5.

We then examined whether manipulating GABA_A_R conformation influences the diffusion of receptors containing the γ2 subunit, which are present at all hippocampal inhibitory synapses and are required for postsynaptic GABA_A_R and gephyrin clustering (Christie et al., 2006; Essrich et al., 1998; Gunther et al., 1995). Neurons were surface-labeled at DIV 21–23 with QD-labeled anti-γ2 antibodies to stain the endogenous protein (see STAR Methods, Bannai et al., 2006; Dahan et al., 2003). Neurons were then exposed to 100 μM muscimol alone or combined with PTX. Cells were imaged between 10 and 50 min after drug application. We found that surface exploration of individual QDs was increased for receptors in both conditions, as compared to control (Figure 1A). Quantitative analysis performed on the bulk (extrasynaptic + synaptic) population of trajectories revealed that the diffusion coefficients of GABA_A_Rγ2 increased by about 40% in muscimol + PTX condition (Figure 1B). Furthermore, the median value of the explored area (EA) increased by about 20% in muscimol + PTX condition (Figure 1C), indicative of decreased receptor confinement when the active state is favored. Instead, application of muscimol alone to promote the desensitized state did not significantly increase the diffusion coefficient of GABA_A_Rγ2 for the bulk population of QDs (Figure 1B) but significantly increased their explored area (Figure 1C). We obtained exactly the same results whether we consider long (50–60 min, ∼10 films, Figures 1B-1C) or short (10–15 min, 2 films, Figures 1D-1E) drug exposure times, indicating that the observed changes in GABA_A_ receptor diffusion are not related to an artifact of long-term imaging or to massive rearrangements of cell signaling (as validated above with calcium imaging).

We then analyzed (considering all movies) the diffusive behavior of GABA_A_Rγ2 in extrasynaptic and synaptic domains of neurons. These domains were visualized by co-transfecting neurons with the inhibitory synaptic marker Gephyrin-FingR-eGFP, which stains endogenous gephyrin proteins (Gross et al., 2013). Application of muscimol and PTX to promote the active state or application of muscimol alone to promote the desensitized state did not significantly increased GABA_A_Rγ2 diffusion coefficient in the extrasynaptic and synaptic membranes (Figures 1F-1H). However, both conditions significantly increased GABA_A_Rγ2 explored area in both synaptic and extrasynaptic domains (Figures 1G–1I). Thus, promoting either GABA_A_R channel active or desensitized states reduced synaptic confinement of GABA_A_Rγ2.

### Promoting GABA_A_R activation or desensitization do not lead to global change in γ2 cluster size and number

We therefore used conventional fluorescence microscopy to examine whether these pharmacological manipulations of GABA_A_Rs altered γ2-subunit membrane clustering in hippocampal neurons. For this purpose, neurons were treated for 2 h with drugs promoting receptor activation or desensitization before fixation and immunostaining for GABA_A_Rγ2 and the Vesicular GABA transporter (VGAT), a marker of presynaptic GABAergic terminals. Neurons were then imaged and the number, surface area and fluorescence intensity of GABA_A_Rγ2 clusters facing presynaptic inhibitory terminals were quantified. In control conditions, GABA_A_Rγ2 formed numerous clusters along the dendrites of neurons facing VGAT-positive inhibitory synaptic boutons (Figure S2A). Pharmacological conditions promoting the active or desensitized conformations of the receptor had no noticeable impact on the density of GABA_A_Rγ2 clusters (Figure S2B). Moreover, no significant difference in the mean cluster size (Figure S2C) or the mean intensity (Figure S2D) was detected in postsynaptic domains, indicating no detectable effect on GABA_A_Rγ2 accumulation at inhibitory synapses. Similarly, the density, size and intensity of extrasynaptic GABA_A_Rγ2 clusters were not significantly affected on drug exposure (Figures S2E-S2G). The density, surface and fluorescence intensity of presynaptic inhibitory boutons labeled for VGAT were also unaffected on application of muscimol (Figures S2H-S2J), indicating no major reorganization of presynaptic boutons. In conclusion, promoting either the active or desensitized states of GABA_A_Rs does not significantly alter the synaptic and extrasynaptic clustering of γ2-containing receptors as tested by conventional fluorescence microscopy.

### Promoting GABA_A_R activation or desensitization lead to gephyrin loss at inhibitory synapses

**Figure 2:**
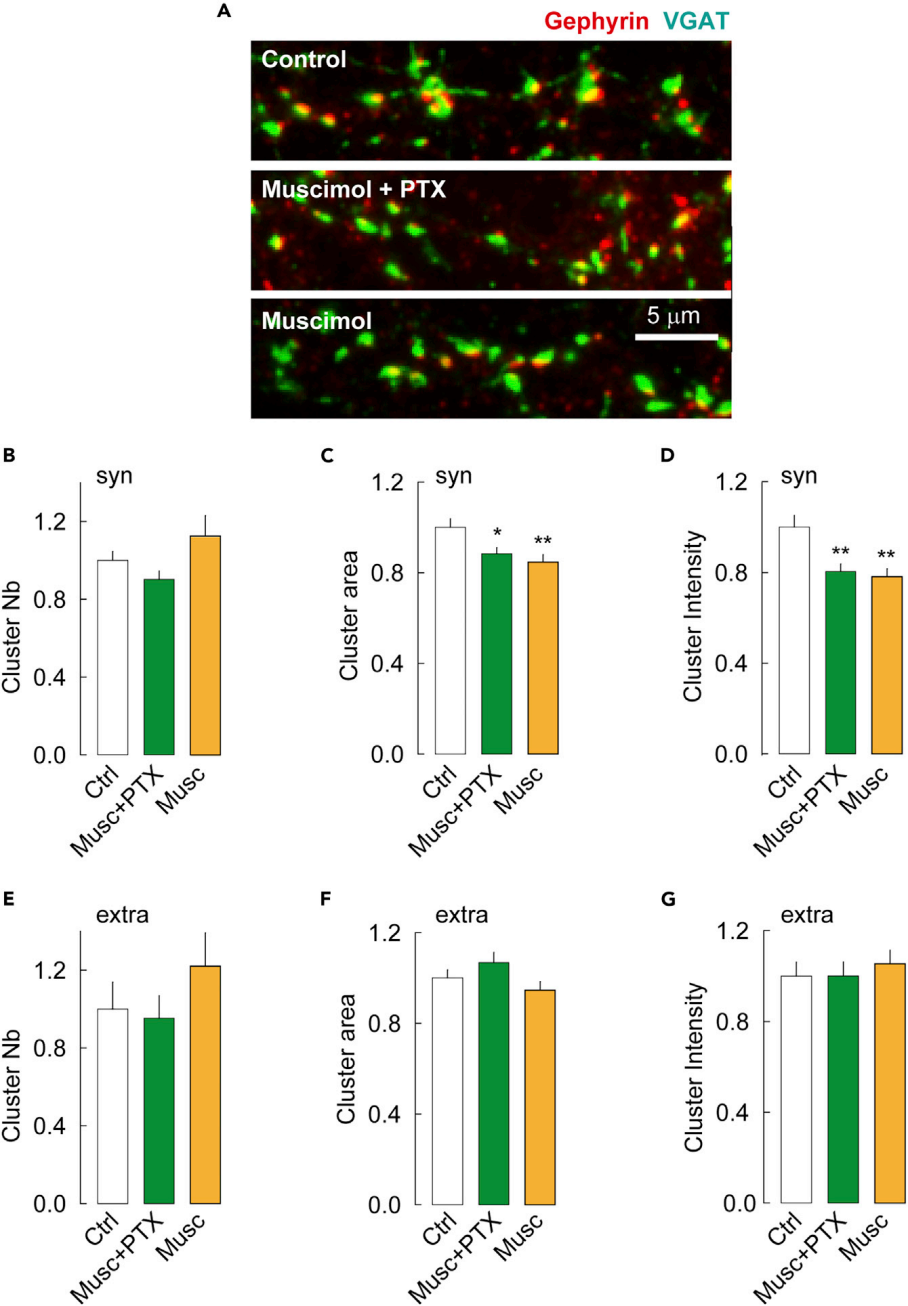
The conformation of the GABA_A_R impairs postsynaptic gephyrin clustering (A) Representative images of hippocampal cultured neurons stained for gephyrin (red), and VGAT (green) and imaged with conventional epifluorescence. Neurons were exposed to drugs (Muscimol + PTX or Muscimol) eliciting the active or desensitized conformational states and compared with control cells. Scale bar, 5 μm. (B–G) Quantification of gephyrin cluster number (B, E), area (C, F), and intensity (D, G) at synapses (B–D) and extrasynaptic sites (E–G) shows that promoting the GABA_A_R active or desensitized conformations reduce the size and intensity of gephyrin clusters at synapses but not at extrasynaptic sites. Ctr, n = 40 cells, Musc + PTX, n = 36 cells, Musc, n = 28 cells, 3 cultures. Synaptic: Musc + PTX, Cluster Nb p = 0.2, area p = 3.0 10^−2^, intensity p = 4.0 10^−3^; MUSC, Cluster Nb p = 0.6, area p < 1.0 10^−3^, intensity p < 1.0 10^−3^; Extrasynaptic: Musc + PTX, Cluster Nb p = 0.6, area p = 0.8, intensity p = 0.9; Musc, Cluster Nb p = 0.2, area p = 0.7, intensity p = 0.2. Data shown as mean ± SEM Values were normalized to the corresponding control values. ∗, p < 5.0 10^−2^; ∗∗, p < 1.0 10^−2^ (Mann–Whitney rank-sum test).

We then determined whether the same manipulations might affect gephyrin clustering. Again using conventional fluorescence microscopy, we observed a reduced immunofluorescence of postsynaptic gephyrin clusters in conditions favoring either the active or desensitized conformations of the receptor, as compared to the control (Figure 2A). Quantitative analysis revealed that although neither treatment favoring active and desensitized GABA_A_R conformations affected the overall number of synaptic gephyrin clusters (Figure 2B), they both reduced gephyrin cluster size by 13 and 21% (Figure 2C) and their fluorescence intensity by 20 and 26% (Figure 2D), respectively. This effect was restricted to synaptic gephyrin clusters since the number, size, and intensity of extrasynaptic clusters were unchanged on treatments (Figures 2E–2G). Altogether, these results indicate that promoting GABA_A_R active and desensitized states reduce gephyrin clustering at inhibitory synapses without significant effect on GABA_A_Rγ2 clustering or presynaptic GABAergic terminals.

### Super-resolution microscopy reveals reorganization of GABA_A_Rγ2 and gephyrin subsynaptic domains on channel activation and desensitization

**Figure 3:**
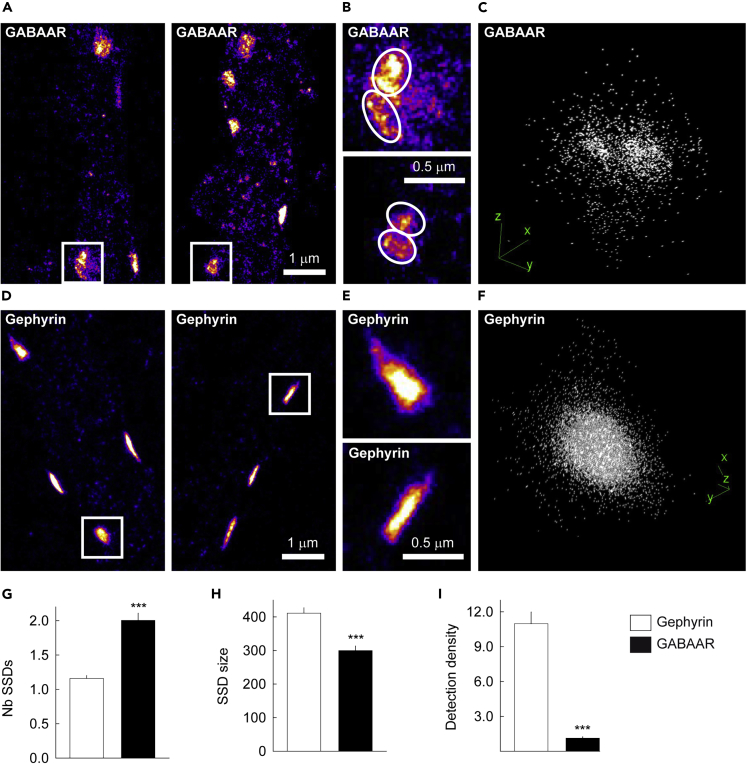
PALM illustrating the nanoscale organization of GABA_A_Rγ2 and gephyrin at inhibitory synapses (A) Representative 2D images of dendritic regions in neurons expressing dendra2-GABA_A_Rγ2. Scale bar, 1 μm. (B) Higher magnification of the regions of interest in A. Scale bar, 0.5 μm. (C) 3D reconstruction of individual synapses showing that GABA_A_Rγ2 form subdomains at the inhibitory synapse. (D) Representative 2D images of dendritic regions in neurons expressing dendra2-gephyrin. Scale bar, 1 μm. (E) Higher magnification of the regions of interest in D. Scale bar, 0.5 μm. (F) 3D images showing the homogeneous distribution of gephyrin at the synapse. (G) Quantification of the number of gephyrin (white) and GABA_A_Rγ2 (black) subdomains per synapse. On average two GABA_A_Rγ2 subdomains are detected per synapse while gephyrin does not form subdomains. (H) Quantification of subdomain size showing larger cluster of gephyrin (white) compared to GABA_A_Rγ2 (black). (I) Quantification of the gephyrin (white) and GABA_A_Rγ2 (black) single molecule detection densities in transfected neurons. Neurons exhibit denser gephyrin packing than GABA_A_Rγ2. G-I: GABA_A_Rγ2, n = 193 subdomains, gephyrin, n = 182 subdomains, 3 cultures. Data are presented as mean ± SEM ∗∗∗, p < 1.0 10^−3^ (Mann–Whitney rank-sum test).

How can it be explained that gephyrin is destabilized at synapses under conditions favoring GABA_A_R activation or desensitization while the receptors are not? Gephyrin and GABA_A_Rs have been shown to stabilize each other at synapses (Essrich et al., 1998; Kralic et al., 2006; Li et al., 2005; Schweizer et al., 2003; Studer et al., 2006). Thus, one might expect that loss of gephyrin would follow receptor loss from the synapse. This is likely here because conformational changes in GABA_A_Rs significantly alter the diffusion and confinement of the γ2 subunit (Figure 1). One possibility is that the effects of drugs on receptor clustering at synapses concern subdomains that are not resolved by conventional epifluorescence microscopy. We thus used nanoscopic imaging to explore the influence of GABA_A_R conformation on its synaptic organization as well as on gephyrin scaffold reorganization. Using photoactivated localization microscopy (PALM), we estimated the spatial resolution to be ∼25–30 nm; hence, image segmentation of the rendered PALM images can resolve substructure organization within a gephyrin or a GABA_A_R cluster that are not discernable using diffraction-limited imaging (Specht, 2019; Specht et al., 2013; Yang and Specht, 2019). Under control conditions, 2D PALM revealed that GABA_A_Rγ2 formed round or elongated structures along the dendrites of the transfected neurons (Figure 3A). A more precise observation of these individual structures revealed the presence of multiple GABA_A_Rγ2 domains separated by zones of weaker or interrupted labeling (Figure 3B). This organization of receptor clusters into subdomains was confirmed by 3D microscopy (Figure 3C). These structures are reminiscent of the SSDs that have been reported in other studies (Crosby et al., 2019; Dzyubenko et al., 2016) and extensively characterized in (Crosby et al., 2019). In contrast, gephyrin clusters showed a more homogeneous and compact domain organization in 2D and 3D PALM (Figures 3D–3F). In these experimental conditions and in agreement with previous studies (Crosby et al., 2019; Dzyubenko et al., 2016; Pennacchietti et al., 2017; Specht et al., 2013), gephyrin clusters containing multiple SSDs were only occasionally observed. These observations were quantified. Cluster analysis on PALM data was done as before (Battaglia et al., 2018). Only clusters of a length ≥50 nm were considered. Quantification showed that, in control conditions, only 10.8% of synapses have multiple gephyrin SSDs while 48.2% have multiple GABA_A_R SSDs. Furthermore, GABA_A_Rγ2 formed on average 2.01 ± 0.10 SSDs, whereas 1.16 ± 0.04 gephyrin SSDs per synapse were detected (Figure 3G). The average surface area of the GABA_A_Rγ2 SSD was 0.030 ± 0.0013 μm^2^while that of gephyrin was 0.068 ± 0.004 μm^2^ (Figure 3H). In addition, the density of detections present within each gephyrin SSD was ∼11 times greater than that detected in the GABA_A_Rγ2 SSDs (Figure 3I). These results show that gephyrin SSDs are larger and more compact than GABA_A_Rγ2 SSDs. Our data are compatible with previous data (Crosby et al., 2019; Pennacchietti et al., 2017; Specht et al., 2013) suggesting that in control condition, gephyrin molecules are tightly grouped to form a compact synaptic platform on which on average two GABA_A_R subdomains are anchored.

**Figure 4:**
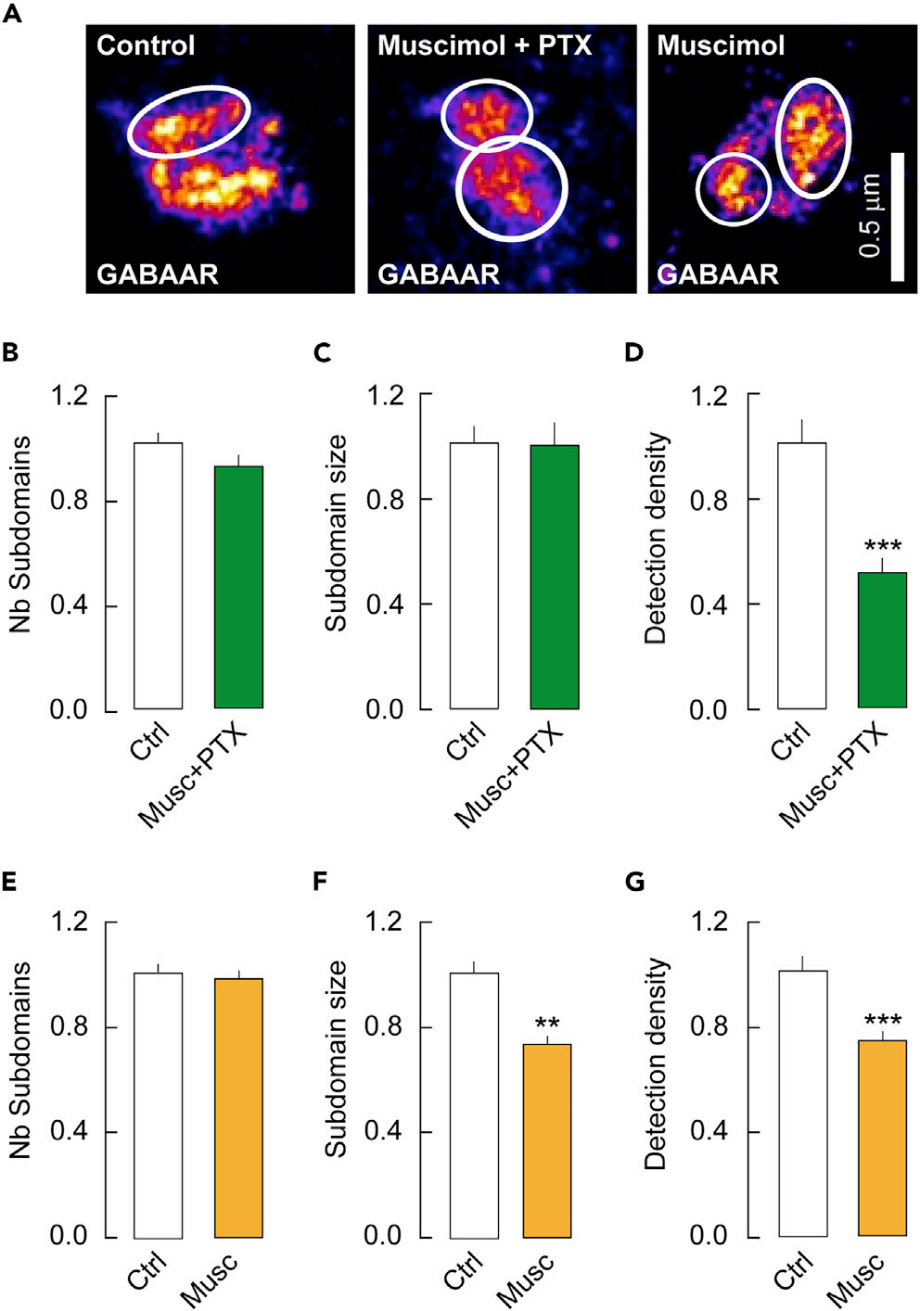
Impact of the conformational states of the GABA_A_R on its organization into subdomains at synapses (A) Representative images of dendra2-GABA_A_Rγ2 in control condition or in the presence of muscimol + PTX or muscimol alone to favor the active and desensitized conformational states. Scale bar, 0.5 μm. Note the reduced labeling in the active and desensitized conformational states of the receptor. (B–D) Quantification of the number (B) of dendra2-GABA_A_Rγ2 subdomains per synapse, the size of SSDs (C) and the density of molecules detected per SSD (D) on muscimol + PTX treatment showing no effect of the drugs on the density of SSDs nor on their size but a significant decrease in the density of single molecules detected per SSD. (B) Ctrl, n = 216 synapses, Musc + PTX, n = 139 synapses, p = 0.82, 3 cultures. (C–D) Ctrl, n = 321 subdomains, Musc + PTX, n = 193 subdomains, (C) p = 0.85, D, p < 1.0 10^−4^, 3 cultures. (E–G) Quantification of the number (E) of dendra2-GABA_A_Rγ2 subdomains per synapse, the size of SSDs (F) and the density of molecules detected per SSD (G) on muscimol treatment showing no effect of the drugs on the density of SSDs but a significant decrease in their size and density of molecules detected per SSD. (E) Ctrl n = 464 synapses, Musc, n = 675 synapses, p = 0.19, 5 cultures. (F–G) Ctrl, n = 330 subdomains, Musc, n = 460 subdomains, (F) p = 1.4 10^−3^, G, p < 1.0 10^−3^, 5 cultures. Data are presented as mean ± SEM ∗, p < 5.0 10^−2^; ∗∗∗, p < 1.0 10^−3^ (Mann–Whitney rank-sum test). See also Figures S2 and S3.

We then estimated the impact of manipulating GABA_A_R conformations on GABA_A_Rγ2 SSDs. Treatments with muscimol alone or in combination with PTX decreased the dendra2-GABA_A_Rγ2 signal (Figure 4A). There was no significant loss of the number of GABA_A_Rγ2 SSDs on application of muscimol + PTX or muscimol alone, respectively (Figures 4B, 4E). Under these different experimental conditions, the remaining GABA_A_Rγ2 SSDs were reorganized. Promoting the active state did not alter the size of the SSDs (Figure 4C) but reduced by 47% the detection density in the SSDs (Figure 4D). Favoring the desensitized state impacted even more the SSDs: it reduced by 25% the size of the SSDs (Figure 4F) and by 24% the detection density in the SSDs (Figure 4G). Therefore, although changes in receptor conformation have little impact on the overall morphology of GABA_A_Rγ2 clusters (Figure S2), high-resolution microscopy reveals a loss of GABA_A_Rγ2 SSDs and a reorganization of the remaining SSDs in the different conformational states.

Because muscimol is not a natural agonist, we wondered whether using the natural agonist GABA would yield similar results on GABA_A_Rγ2 SSDs. To induce the desensitized conformation, we used GABA at 1 mM. To elicit the open conformation, we used GABA at 1 and 10 mM in combination with PTX. We tested two different concentrations of GABA because PTX decreases the apparent affinity of the receptor for GABA (Gielen and Corringer, 2018). Indeed, if we are not saturating in GABA + PTX condition, there might be a non-negligible population of receptors in the resting state. Our results show that GABA + PTX or GABA treatments alter the morphological organization of GABA_A_Rγ2 SSDs whatever the concentration of GABA (1 or 10 mM) (Figure S3A). Concerning the GABA + PTX condition, we observed a decrease in the density of molecules per SSD (Figure S3B) without any change in the number of SSDs or their size (Figures S3C, S3D respectively) whatever the concentration of GABA used (1 and 10 mM). This effect is identical to that observed in muscimol + PTX condition (Figures 4B-4D). The GABA condition to elicit GABA-induced receptor desensitization decreases the number of SSDs (Figure S3E) without changing the size (Figure S3F) or density (Figure S3G) of molecules per SSD. This result differs from the muscimol condition for which the size and density of molecules per SSD were decreased while the number of SSDs remained unchanged (Figures 4E–4G). In both experimental conditions however, the changes result in a loss of GABA_A_Rγ2 at the synapse and are therefore consistent with each other. This allows us to conclude that our experimental conditions are robust and reproducible whatever the GABA_A_ receptor agonist used.

**Figure 5:**
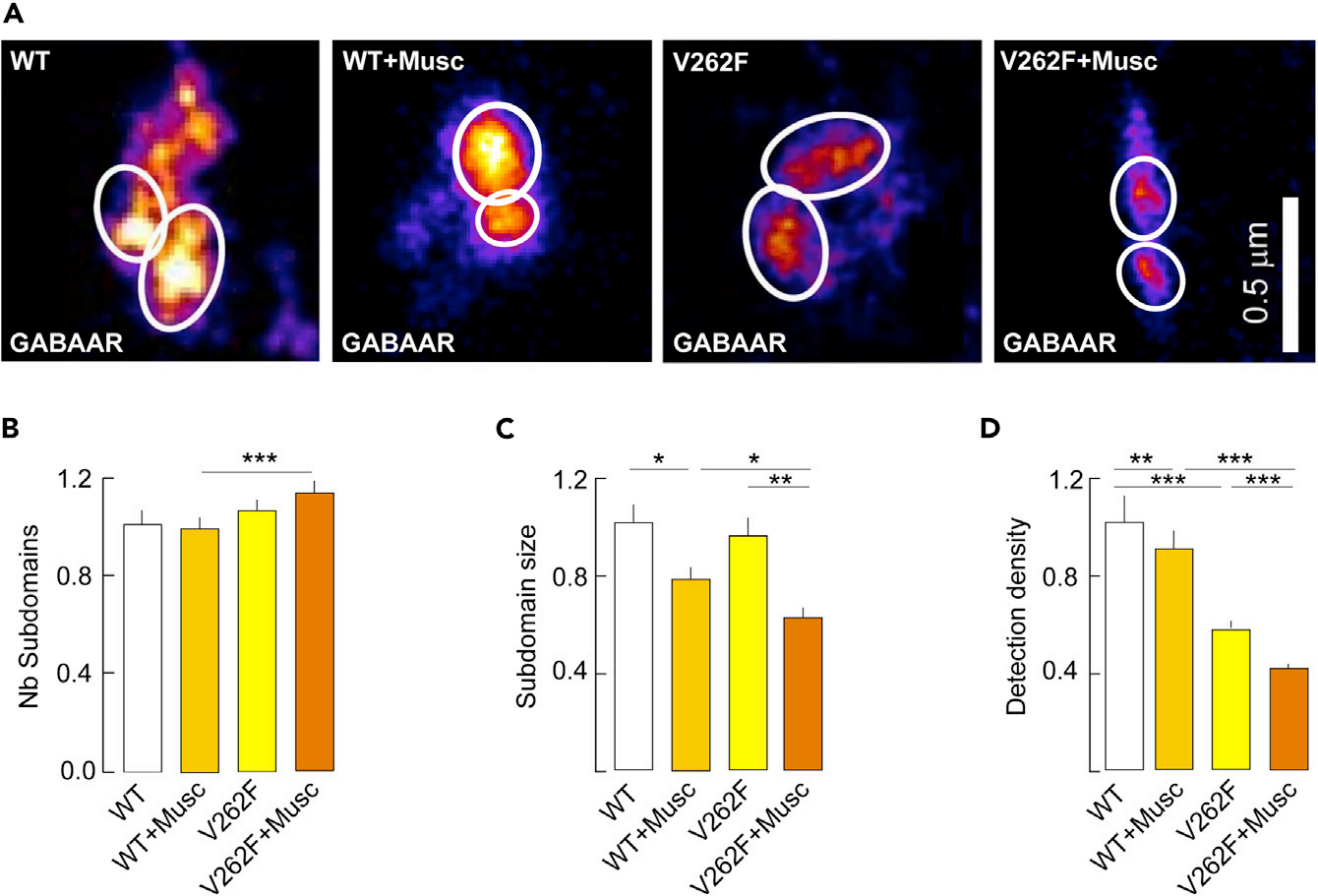
Impact of the γ2^V262F^mutation on GABA_A_R subsynaptic domains and response to muscimol (A) Representative images of dendra2-γ2^WT^ and dendra2-γ2^V262F^ in neurons transfected at DIV14 and imaged at DIV21 in control conditions or in the presence of muscimol. Scale bar, 0.5 m. Note the reduced GABA_A_Rγ2 labeling for the γ2^V262F^mutant versus the γ2^WT^. (B–D) Quantification of the number of dendra2 subdomains per synapse (B), the SSD size (C) and the density of molecules detected per SSD (D) showing in control condition an important loss of GABA_A_Rγ2 molecules per SSD for the γ2^V262F^mutant as compared to the γ2^WT^. Note also the greater impact of the muscimol treatment on SSD size and detection density for γ2^V262F^ as compared to γ2^WT^. (B) γ2^WT^, n = 114 synapses, γ2^V262F^, n = 121 synapses, γ2^WT^ + Musc, n = 204 synapses, γ2^V262F^ + Musc, n = 156 synapses, γ2^WT^ versus γ2^V262F^, p = 0.38, γ2^WT^ versus γ2^WT^ + Musc, p = 0.11, γ2^WT^ + Musc versus γ2^V262F^ + Musc, p < 1.0 10^−3^, γ2^V262F^ versus γ2^V262F^ + Musc, p = 0.46, 2 cultures. (C–D) γ2^WT^, n = 165 subdomains, γ2^V262F^, n = 193 subdomains, γ2^WT^ + Musc, n = 301 subdomains, γ2^V262F^ + Musc, n = 264 subdomains. (C) γ2^WT^ versus γ2^V262F^, p = 0.58, γ2^WT^ versus γ2^WT^ + Musc, p = 0.04, γ2^WT^ + Musc versus γ2^V262F^ + Musc, p = 0,02, γ2^V262F^ versus γ2^V262F^ + Musc, p = 7.0 10^−3^ (D) γ2^WT^ versus γ2^V262F^, p < 1.0 10^−3^, γ2^WT^ versus γ2^WT^ + Musc, p = 1.3 10^−3^, γ2^WT^ + Musc versus γ2^V262F^ + Musc, p < 1.0 10^−3^, γ2^V262F^ versus γ2^V262F^ + Musc, p < 1.0 10^−3^Data are presented as mean ± SEM ∗, p < 5.0 10^−2^; ∗∗, p < 1.0 10^−2^, ∗∗∗, p < 1.0 10^−3^ (Mann–Whitney rank-sum test).

We then used mutagenesis to quantify the muscimol effect on receptors displaying highly enhanced desensitization. We expressed the GABA_A_Rγ2L^V262F^mutant, which has been shown in the presence of agonist to increase α1β2γ2L receptor desensitization rate by ∼12-fold in *Xenopus laevis* oocytes, and yields GABA_A_Rs that fully desensitize (steady-state current equating ∼1% of the peak current under desensitizing conditions) with minimal effect on receptor gating (Gielen et al., 2015). In the present neuronal culture, this mutant is thus predicted to undergo more profound agonist-elicited desensitization than the wildtype. Neurons were transfected at DIV14 with recombinant, wild-type (γ2L^WT^) or mutant (γ2L^V262F^) dendra2-γ2L and imaged at DIV21. Using PALM, we observed in absence of drugs a reduction in dendra2 labeling in neurons that expressed γ2L^V262F^ compared to neurons expressing γ2L^WT^(Figure 5A). Quantification indicated a 58% decrease in the density of single molecules detected per SSD in γ2L^V262F^ versus γ2L^WT^ expressing neurons without no change in the number of SSDs per synapse or their size (Figures 5B–5D). We then promoted desensitization with muscimol and compared its impact on γ2L^WT^ versus γ2L^V262F^ SSDs. Favoring desensitization had no significant effect on the number of SSDs per synapse in either γ2L^WT^ or γ2L^V262F^ transfected neurons (Figure 5B). In contrast, muscimol exposure significantly decreased the size of SSDs and the detection density per SSD for both γ2L^WT^ and γ2L^V262F^(Figures 5C and 5D). However, muscimol more strongly impacted SSDs in neurons expressing γ2L^V262F^ than γ2L^WT^, consistent with the gain-of-desensitization phenotype of the V262F mutation. Indeed, muscimol treatment induced a 21 and 8% decrease in SSD size and intra-SSD molecule density in neurons transfected with γ2L^WT^ whereas the decrease was 37 and 22% in neurons transfected with γ2L^V262F^(Figures 5C and 5D). Furthermore, the fact that under resting conditions the molecular density of SSDs from γ2L^V262F^-expressing neurons is significantly smaller than that of γ2L^WT^-expressing neurons (Figure 5D) allows us to hypothesize that spontaneous neuronal release of GABA is sufficient to desensitize the mutant receptor.

**Figure 6:**
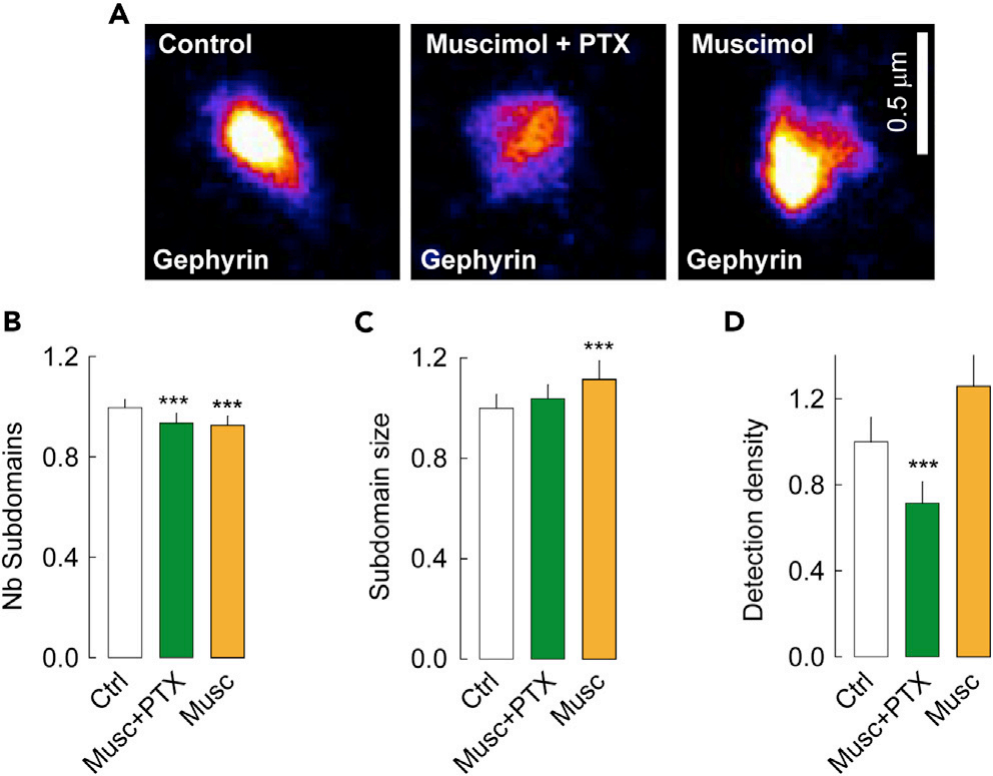
GABA_A_R conformational states regulate the nanoscale distribution of gephyrin at inhibitory synapses (A) Representative images of dendra2-gephyrin in the presence of muscimol + PTX or muscimol to elicit the active and desensitized conformational states of the GABA_A_R, respectively. Scale bar, 0.5 μm. (B) Reduced number of gephyrin subdomains per synapse when favoring the active and desensitized receptor conformational states. Ctrl, n = 157 synapses, Musc + PTX, n = 176 synapses, p < 1.0 10^−3^, Musc, n = 136 synapses, p < 1.0 10^−3^, 3 cultures. (C) No effect of the active and desensitized GABA_A_R conformations on gephyrin subdomain size, as compared to controls. Ctrl, n = 182 subdomains, Musc + PTX, n = 199 subdomains, p = 0.7, Musc, n = 154 subdomains, p = 0.6. (D) Quantification of the density of detection per square micrometer in transfected neurons. The open conformation of GABA_A_R reduces the packing of gephyrin at synapses. Musc + PTX, p < 1.0 10^−3^, Musc, p = 0.7. Data are presented as mean ± SEM ∗∗∗, p < 1.0 10^−3^ (Mann–Whitney rank-sum test). See also Figure 2.

PALM imaging further revealed that gephyrin SSDs were also affected (Figure 6A). Promoting receptor active state with muscimol + PTX reduced the number of gephyrin SSDs per synapse and the density of detections per SSD by 6 and 29%, respectively (Figures 6B, 6D) with no effect on their size (Figure 6C). Altogether, these data report a loss of gephyrin molecules at inhibitory synapses in conditions favoring GABA_A_R active state. Promoting receptor desensitization with muscimol alone on the other hand significantly reduced the number of gephyrin SSDs per synapse by 7% (Figure 6B). The remaining SSDs were not significantly changed in size and in detection density (Figures 6C and 6D). Together, our QD-SPT and PALM results indicate that favoring the active or desensitized states of the GABA_A_R affects the diffusion-capture of the receptor at the synapse, thereby leading to a reorganization of receptor SSDs and a loss of gephyrin.

### The desensitized GABA_A_Rs are confined within endocytic zones

We then tested the hypothesis that desensitized receptors depleted from the synapse are confined in endocytic zones for storage or internalization. For this, we analyzed using QD-SPT the diffusion of GABA_A_Rγ2 in endocytic regions identified by the presence of clathrin-YFP in transfected neurons. As illustrated in Figure S4A, individual GABA_A_Rγ2 trajectories exhibited a decrease in surface exploration in clathrin-YFP clusters in conditions favoring receptor desensitization. This was consistent with the observed decrease in diffusion coefficients (although not significant, p = 0.07) of GABA_A_Rγ2 in endocytic zones (Figure S4B). Quantification of explored area in endocytic zones showed a significant decrease (Figure S4C). Therefore, our data support that desensitized receptors are confined within endocytic wells.

**Figure 7:**
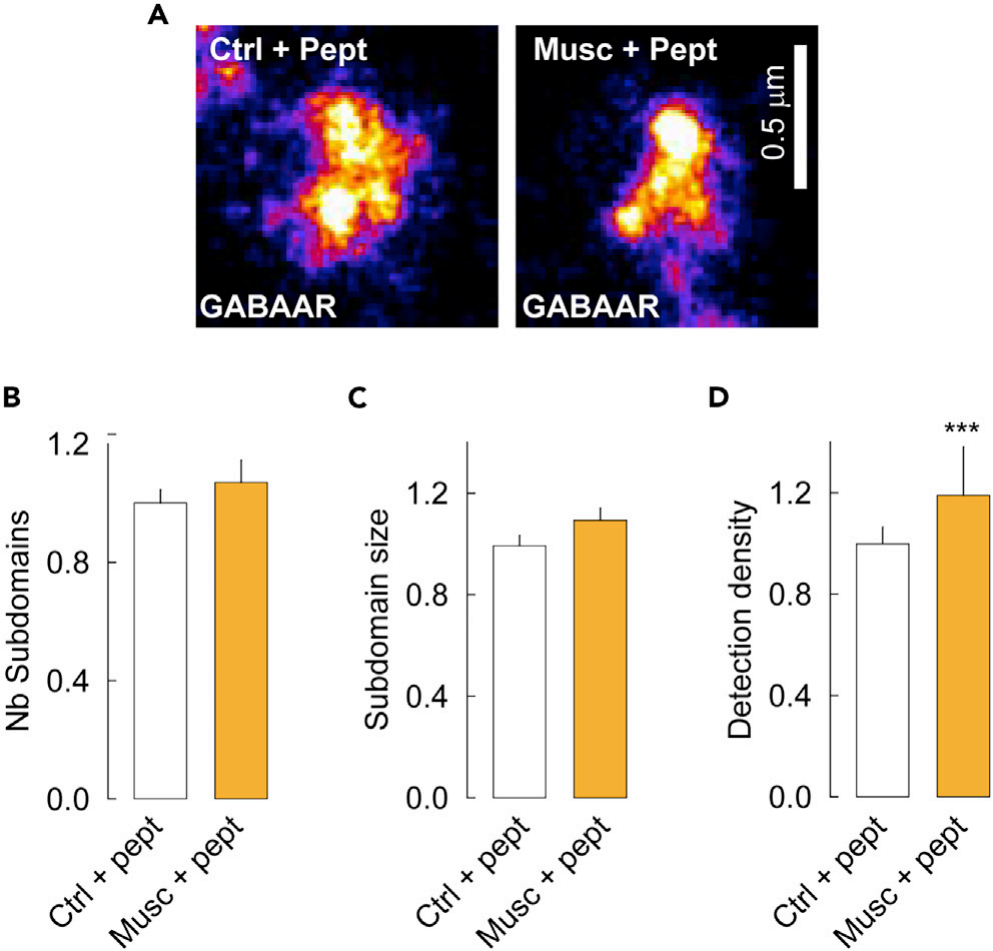
Blockade of clathrin-mediated endocytosis prevents reorganization of GABA_A_Rγ2 subdomains induced by the desensitized states (A) Representative images of dendra2-GABA_A_Rγ2 in control versus desensitized conformational states under conditions of clathrin-mediated blockade of endocytosis. Scale bar, 0.5 μm. (B–D) Impact of blocking endocytosis on the number (B), size (C) and single molecule detection density (D) of receptor subdomains. Note the absence of effect of the desensitized conformation promoted by muscimol treatment on subdomain number and size on blockade of clathrin-mediated endocytosis. Furthermore, receptor desensitization increased the density of molecules detected within SSDs as compared to the control condition. (B) Ctrl, n = 280 synapses, Musc, n = 248 synapses, p = 0.3, 3 cultures. (C–D) Ctrl, n = 532 subdomains, Musc, n = 475 subdomains, (C) p = 0.4, (D) p < 1.0 10^−3^, 3 cultures. Data are presented as mean ± SEM ∗, ∗∗∗, p < 1.0 10^−3^ (Mann–Whitney rank-sum test). See also Figures S4 and S7.

In agreement with these observations, PALM revealed that the reorganization of the GABA_A_Rγ2 SSDs observed under conditions promoting desensitization was mainly prevented by inhibiting clathrin-mediated endocytosis with a blocking peptide (Figure 7A). Quantitative analysis confirmed these observations. The blocking peptide prevented the desensitization-induced decrease in the size and density of molecules detected per SSD (Figures 7B–7D) observed in absence of peptide (Figures 4F and 4G). Conversely, the molecular density per SSD was significantly increased in neurons in which the desensitized state was favored in the presence of the endocytosis blocking peptide (Figure 7D), indicating receptor accumulation. Therefore, we propose that desensitized receptors are captured in endocytic zones where they are internalized.

### The GABA_A_R conformational states alter the synaptic diffusion and clustering of the synaptic α1 subunit

Because the γ2 subunit is present in most synaptic and extrasynaptic GABA_A_Rs of hippocampal neurons (Essrich et al., 1998), we asked whether promotion of different conformational states of GABA_A_Rs might influence in particular a subunit enriched at inhibitory synapses. We therefore investigated the impact of pharmacological manipulations on the lateral diffusion of the α1 subunit, which is concentrated at a subset of inhibitory synapses (i.e., those containing α1β2/3γ2 heteropentamers) (Brünig et al., 2002). Compared with the control condition, promoting the active conformation of the receptor with muscimol + PTX increased the length of α1 trajectories while favoring the desensitized conformation with muscimol alone shortened them (Figure S5A). Treatment with muscimol + PTX significantly increased the diffusion coefficient (Figure S5B) and explored area (Figure S5C) of α1-containing GABA_A_Rs for the whole (synaptic + extrasynaptic) population of QDs, indicating reduced confinement. This effect on the overall population of QDs in fact reflected that of the extrasynaptic receptors, which were strongly accelerated (Figure S5D) and poorly confined (Figure S5E), compared to receptors in control conditions. In contrast, this treatment slowed down α1-containing receptors at synapses (Figure S5F) and significantly increased their confinement (Figure S5G). This result differed from those obtained with the γ2 subunit where both extrasynaptic and synaptic QDs were free to diffuse on channel activation (Figures 1G, 1I). Promoting receptor desensitization with muscimol alone instead had no effect on the mean diffusion coefficients of α1-containing receptors for either bulk, synaptic or extrasynaptic QDs (Figures S5B, S5D, S5F). In contrast, the explored area was considerably reduced for all QDs (Figures S5C, S5E, S5G). Therefore, promoting receptor desensitization increased the synaptic confinement of the α1-containing receptors. This effect is opposite to that observed for γ2-containing receptors (Figure 1), whose synaptic mobility was increased.

The increased synaptic confinement of α1-containing receptors in conditions promoting the active or desensitized states was correlated with a reduction in the number of synaptic clusters per dendritic length (Figures S6A-S6B), indicating a loss of GABA_A_Rs containing the α1 subunit at inhibitory synapses. The synaptic clusters of the α1 subunit, which persisted at other synapses, were not significantly altered in size and intensity (Figures S6C-S6D). These results differ from those obtained for the γ2 subunit showing no changes in cluster density and receptor amount at inhibitory synapses with conventional epifluorescence (Figure S2). It is only with PALM imaging that a significant loss of the γ2 subunit was reported at inhibitory synapses (Figure 4).

The significant decrease in clustering of the α1 subunit (Figure S6) and not of the γ2 subunit (Figure S2) observed with conventional microscopy prompted us to assess changes in the overall membrane pool of the α1 subunit with epifluorescence to determine whether the treatments altered receptor amount at the cell surface. Quantification of the average intensity per pixel of α1 subunit surface labeling reveals a 22 and 20% reduction in the α1 membrane pool when forcing open or desensitized conformations of the receptor under muscimol + PTX and muscimol conditions, respectively. Furthermore, we show that this is dependent on clathrin-mediated endocytosis because, in the same cultures, the effect of the drugs (Figures S7A and S7B) was blocked in the presence of an inhibitory peptide (Figures S7C-S7D). These results, together with SPT data showing confinement of the desensitized receptor in endocytic wells (Figure S4) and super-resolution imaging showing no reorganization of SSDs in the desensitized condition in the presence of an inhibitory peptide for endocytosis (Figure 7) allow us to propose that desensitized GABA_A_ receptors are internalized.

Altogether our data report that although promoting receptor active or desensitized conformations had different effects on the mobility of the γ2 and α1 subunit of the GABA_A_R at synapses (with γ2-containing receptors being less confined), both conditions do, however, result in the synaptic loss of receptors containing γ2 and α1 subunits, most likely reflecting their internalization at perisynaptic endocytic wells. Furthermore, our results suggest that the impact of favoring GABA_A_R active and desensitized states is greater on α1-than on non α1-containing receptors.

## Discussion

Negative and positive allosteric modulators of the GABA_A_R respectively decrease and increase the mobility of GABA_A_Rs (Gouzer et al., 2014; Lévi et al., 2015). These results revealed that the diffusion of GABA_A_Rs is sensitive to the conformational changes induced by benzodiazepines. Here, we aimed to test the possibility that diffusion is also sensitive to conformational changes promoted by agonists associated with the active and desensitized states. Our results show that promoting either conformation alters the membrane dynamics and synaptic accumulation of GABA_A_R containing γ2 and α1 subunits. This effect is accompanied by the destabilization of the synaptic gephyrin scaffold.

### Short-term versus long-term effects of GABA_A_R activation and blockade on receptor diffusion

Gouzer et al. (2014) proposed that there is an adaptive regulation of GABAergic synapses through regulation of receptor synaptic diffusion and clustering in response to GABA_A_R activation or inhibition. In their study, muscimol increases the diffusion of the GABA_A_R α2 subunit while decreasing GABA_A_Rα2 and gephyrin levels at synapses. These effects of the agonist were observed 30 to 120 min after treatment. These observations are similar to our results on the effect of muscimol on the synaptic organization of the GABA_A_R γ2 subunit and of gephyrin. The effect of muscimol-induced GABA_A_R desensitization on receptor density at synapses was potentiated in the γ2L^V262F^mutant suggesting that at least some of the effects of muscimol observed on GABA_A_R and gephyrin in the study by Gouzer et al. (2014) were because of receptor desensitization.

### Impact of receptor activation and desensitization on gephyrin and GABA_A_R SSDs

Our PALM analysis of hippocampal GABAergic inhibitory synapses is in agreement with published work showing that GABA_A_R and gephyrin form SSDs at synapses (Crosby et al., 2019; Dzyubenko et al., 2016; Pennacchietti et al., 2017; Specht et al., 2013). These SSDs are closely associated with presynaptic RIM proteins (Crosby et al., 2019). This synapse organization suggests the existence of *trans*-synaptic nanocolumns, as the ones identified at excitatory glutamatergic synapses (Haas et al., 2018), that play an important role in regulating synaptic transmission (discussed in Yang and Specht, 2019). In agreement with Crosby et al. (2019), we found that under basal activity condition, synapses containing multiple GABA_A_R SSDs are more frequent than the one containing multiple gephyrin SSDs. In our experimental conditions, only 10.8% of synapses have multiple gephyrin SSDs while 48.2% of synapses have several GABA_A_R SSDs. We found an average of 2.01 receptor SSDs/synapse and 1.16 gephyrin SSDs/synapse. Furthermore, the density of detection of gephyrin molecules per SSD is approximately 11 times that of GABA_A_R, indicating greater concentration of gephyrin molecules at postsynaptic site. These results are in favor of the existence of a compact and dense lattice of gephyrin molecules under the synapse capable of recruiting receptors organized into SSDs. An excess of gephyrin molecules at the synapse may allow recruiting in a fast manner additional receptors by diffusion-capture e.g. in conditions of synaptic potentiation.

The induction of inhibitory long-term potentiation induces the formation of newly generated gephyrin SSDs at a subset of inhibitory synapses (Pennacchietti et al., 2017). Conversely, we show that the receptor active and desensitized conformations decrease the number of gephyrin SSDs at inhibitory synapses. This conformation-induced loss of gephyrin SSDs was associated with a reorganization of gephyrin SSDs. Inducing the active conformation of the receptor reduced the density of gephyrin molecules detected per SSD without any change on the size of these SSDs, indicating a scattering of scaffolding proteins per SSD. Favoring the desensitized conformation of the GABA_A_R in contrast increased the size of gephyrin SSD without significant change in the detection density. This reorganization of gephyrin SSDs induced by favoring the active and desensitized conformations of the GABA_A_R was accompanied by a reorganization of GABA_A_R SSDs which were decreased in size and detection density, respectively. Our results, therefore, report a disorganization of the SSDs at inhibitory synapses, which could be responsible for an alteration of synaptic transmission. Our data further show that the loss of receptors at synapses induced by the active and desensitized states is associated with a loss in gephyrin. Thus, our hypothesis is that a conformational change in the receptor is transmitted to gephyrin and reduces gephyrin-receptor interaction, as has been shown elsewhere (Battaglia et al., 2018).

We evaluated the effects of GABA_A_R activation and desensitization on the mobility and aggregation of two different subunits: the γ2 subunit, found in both extrasynaptic and synaptic receptors, and the α1 subunit found mainly at synapses. We found that activation and desensitization had different impacts on the diffusion of receptors containing the γ2 and α1 subunits. The synaptic confinement of γ2 was reduced while that of α1 was increased. However, these different diffusion behaviors resulted in a loss in γ2 and α1 subunits at synapses. We also noticed that the impact of conformation on receptor clustering is significantly stronger on the α1 subunit than on the γ2 subunit of GABA_A_R. Indeed, a loss in α1 subunit at synapses was observed with standard epifluorescence, whereas a loss of the γ2 subunit could only be detected with PALM. This difference could be explained by the fact that the γ2 subunit is present in synaptic receptors containing either α1, α2 or α4 subtypes. Knowing that these subunits have different mobilities at synapses (Hannan et al., 2020), our data suggest that activation or desensitization impacts receptors composed of distinct α subunits differently.

### The endocytic region as a recovery zone of the resting conformation of the receptor

We showed that on desensitization, the scattering of receptors within SSDs was associated with an increased confinement within perisynaptic endocytic zones. Moreover, alterations of GABA_A_R and gephyrin SSDs at synapses induced by the desensitized state could be prevented on blockade of clathrin-mediated endocytosis with an inhibitory peptide. These results allowed us to propose that desensitized receptors are confined within perisynaptic endocytic zones to be internalized and recycled or degraded. However, it is possible that desensitized receptors could be released from endocytic regions once they have returned to a resting conformation. Endocytic zones have been shown in the case of AMPA receptors to constitute a reservoir of receptors that can be delivered to synapses on request (Petrini et al., 2009). QD-SPT experiments reported that GABA_A_Rs are also highly confined within endocytic regions (Smith et al., 2012). We propose on the basis of our results that endocytic regions would be transition zones where active or desensitized receptors are confined waiting to return to a resting conformation and participate again in synaptic transmission.

### Role of GABA_A_R desensitization in synaptic transmission

It has been proposed for AMPARs at glutamatergic synapses (Constals et al., 2015) and for GABA_A_Rs at inhibitory synapses (deLuca et al., 2017) that the exchange of desensitized synaptic receptors with resting ones by lateral diffusion reduces the amount of synaptic desensitization. Our results support the hypothesis that the level of synaptic desensitization is partially controlled by lateral diffusion of GABA_A_Rs. Following agonist binding and activation, desensitized receptors diffuse out of synapses and are captured by perisynaptic endocytic zones. The receptors would be stored transiently in the endocytic zones until they go back to their resting conformation. Resting receptors would then return to synapses where they could participate again to synaptic transmission. During high-frequency stimulation, we propose that desensitized GABA_A_Rs are internalized and sent to degradation. This would be a way to maintain the fidelity of synaptic transmission (Constals et al., 2015) or even increase it (Field et al., 2022) by allowing the synchronized delivery of resting receptors, ready to open and induce ion flow.

### Limitations of the study

All results were used for analysis except in few cases. Cells with signs of suffering (apparition of blobs, fragmented neurites) were discarded from the analysis.

## STAR★Methods

### Key resources table

**Table:** 

REAGENT or RESOURCE	SOURCE	IDENTIFIER
**Antibodies**		

GABA_A_R γ2 subunit (guinea pig)	Synaptic Systems	Cat#224 004; RRID:AB_10594245
GABA_A_R γ2 subunit (rabbit)	Synaptic Systems	Cat#224 003; RRID:AB_2263066
GABA_A_R α1 subunit	Synaptic Systems	Cat#224 203; RRID:AB_2232180
VGAT	Synaptic Systems	Cat#131 011; RRID:AB_887872
Goat antiMouse FITC	Jackson Immunoresearch	Cat#115-095-003; RRID:AB_2338589
Goat antiRabbit CY3	Jackson Immunoresearch	Cat#111-095-003; RRID:AB_2337972
Donkey antiGuinea pig CY3	Jackson Immunoresearch	Cat#706-165-148; RRID:AB_2340460
Goat antiGuinea pig biotinylated Fab	Jackson Immunoresearch	Cat#106-066-003; RRID:AB_2337413
Goat antiRabbit biotinylated Fab	Jackson Immunoresearch	Cat#111-067-003; RRID:AB_2337971
streptavidin-coated quantum dots	Invitrogen	Cat#Q10123MP
anti-rabbit F(ab’)^2^-QDs 655 nm	Invitrogen	Cat#10592815
Fluo4AM	Life Technologies	Cat#14217

**Chemicals, peptides, and recombinant proteins**		

Transfectin	BioRad	Cat#1703351
MCPG	HelloBio	Cat#HB0056
kynurenic acid	Abcam	Cat#ab120256
TTX	HelloBio	Cat#HB1034
Muscimol	HelloBio	Cat#HB0887
Picrotoxin	Abcam	Cat#ab120315
GABA	Sigma	Cat#A2129
NMDA	HelloBio	Cat#HB0454

**Experimental models: Organisms/strains**		

Rat, Sprague Dawley	JanvierLabs	RRID: RN-SD-F

**Recombinant DNA**		

EYFP-Clathrin	Rust et al. (2004)	Addgene Cat#20921
pCAG_GPHN.FingR-eGFP-CCR5TC	Gross et al. (2013)	Addgene Cat#46296
dendra2-GABA_A_Rγ2^WT^	Battaglia et al. (2018)	N/A
dendra2-gephyrin	Battaglia et al. (2018)	N/A
dendra2-GABA_A_Rγ2^V262F^	This paper	N/A

**Software and algorithms**		

MetaMorph	Roper Scientific	http://www.moleculardevices.com/pages/software/metamorph.html
MetaView	Meta Imaging 7.7	https://meta-imaging-series.software.informer.com/
ImageJ	National Institutes of Health and LOCI, University of Wisconsin	https://imagej.net
MATLAB	The Mathworks	https://www.mathworks.com/
SigmaPlot 12.5	Systat Software	http://www.sigmaplot.co.uk/
NIS Elements	Nikon	https://www.microscope.healthcare.nikon.com/products/software

### Resource availability

#### Lead contact

Any additional information or enquiries about reagents and resources should be directed to the Lead contact, Sabine Lévi (sabine.levi@inserm.fr).

#### Materials availability

The transfer of plasmids generated for this study will be made available upon request. A Materials Transfer Agreement may be required.

### Experimental model and subject details

For all experiments performed on primary cultures of hippocampal neurons, animal procedures were carried out according to the European Community Council directive of 24 November 1986 (86/609/EEC), the guidelines of the French Ministry of Agriculture and the Direction Départementale de la Protection des Populations deParis (Institut du Fer à Moulin, Animalerie des Rongeurs, license C 72-05-22). All efforts were made to minimize animal suffering and to reduce the number of animals used. Timed pregnant Sprague-Dawley rats were supplied by Janvier Lab and embryos were used at embryonic day 18 or 19 as described below.

#### Dissociated hippocampal cultures

Primary cultures of hippocampal neurons were prepared as previously described (Chamma et al., 2013). Briefly, hippocampi were dissected from embryonic day 18 or 19 Sprague-Dawley rats of either sex. Tissue was then trypsinized (0.25% v/v), and mechanically dissociated in 1X HBSS (Invitrogen, Cergy Pontoise, France) containing 10 mM HEPES (Invitrogen). Neurons were plated at a density of 140 × 10^3^ cells/mL onto 18-mm diameter glass coverslips (Assistent, Winigor, Germany) pre-coated with 50 μg/mL poly-D,L-ornithine (Sigma-Aldrich, Lyon, France) in plating medium composed of Minimum Essential Medium (MEM, Invitrogen) supplemented with horse serum (10% v/v, Invitrogen), L-glutamine (2 mM) and Na^+^ pyruvate (1 mM) (Invitrogen). After attachment for 3–4 h, cells were incubated in culture medium that consists of Neurobasal medium (Invitrogen) supplemented with B27 (1X) (Invitrogen), L-glutamine (2 mM) (Invitrogen), and antibiotics (penicillin 200 units/mL, streptomycin, 200 μg/mL) (Invitrogen) for up to 4weeksat 37°C in a 5% CO_2_ humidified incubator. Each week, one-third of the culture medium volume was renewed.

### Method details

#### DNA constructs

The following constructs were used: EYFP-Clathrin (Rust et al., 2004) was a gift from Xiaowei Zhuang (Addgene plasmid # 20921; http://n2t.net/addgene:20921; RRID:Addgene_20921), pCAG_GPHN.FingR-eGFP-CCR5TC (Gross et al., 2013) was a gift from Don Arnold (Addgene plasmid # 46296; http://n2t.net/addgene:46296; RRID:Addgene_46296), dendra2-GABA_A_Rγ2^WT^ and dendra2-gephyrin were previously characterized and used (Battaglia et al., 2018). dendra2-GABA_A_Rγ2^V262F^ was mutated from dendra2-GABA_A_Rγ2^WT^ construct.

#### Neuronal transfection

Transfections were carried out at DIV 13–14 using Transfectin (BioRad, Hercules, USA), according to the manufacturers’ instructions (DNA:transfectin ratio 1 μg:3 μL), with 0.5–1 μg of plasmid DNA per 20 mm well. Experiments were performed 7 to 10 days post-transfection.

#### Pharmacology

Experiments were carried out in conditions of neurotransmitter release and glutamatergic activity blockade by applying acutely the metabotropic glutamate receptor antagonist (S)-α-methyl-4-carboxyphenyl-glycine (MCPG; 500 μM; HelloBio, Bristol, UK), the ionotropic glutamate receptor antagonist 4-hydroxy- quinoline-2-carboxylic acid (kynurenic acid; 1 mM; Abcam, Cambridge, UK) and the voltage-gated sodium channels blocker tetrodotoxin (TTX; 1 μM; HelloBio, Bristol, UK). The active state of the GABA_A_R was favored by adding the GABA_A_R selective agonist muscimol (100 μM; HelloBio, Bristol, UK) or GABA (1 mM or 10 mM, Sigma) in the presence of the GABA_A_R channel blocker picrotoxin (PTX, 500 μM; Abcam, Cambridge, UK). The desensitized state was favored using a saturating amount of muscimol (100 μM) or GABA (1mM).

For single particle tracking experiments, neurons were transferred to a recording chamber and were pre-incubated for 5 min at 33°C with the drugs directly added to the imaging medium before starting the recordings. The imaging medium contains MEM without phenol red (Invitrogen) to limit the auto-fluorescence and is supplemented with glucose (33 mM; Sigma), HEPES (20 mM) (Invitrogen), glutamine (2 mM) (Invitrogen), sodium pyruvate (1 mM) (Invitrogen) and B27 (1X) (Invitrogen). For calcium imaging, cells were loaded with Fluo4-AM (Life Technologies), transferred to a recording chamber, imaged in imaging medium first in absence of drugs for 300 s and then in presence of the appropriate drugs for another 600 s. For the immunocytochemistry, drugs were added to the culture medium 2 h prior fixation, in an incubator at 5% CO_2_ and at 37°C.

In some experiments, the formation of endocytic zones was prevented by disrupting the interaction between dynamin and amphiphysin, an interaction that is essential for clathrin-coated pit-mediated endocytosis. The blockade of the endocytic zones was performed in neurons labeled for the γ2 subunit. For this purpose, cultured hippocampal neurons were pre-incubated in imaging medium with a 10 amino acid peptide (25 μM) (R&Dsystems) that blocks endocytosis together with drugs eliciting the desensitized conformational state.

#### Live cell staining for single particle tracking

Neurons were stained as described previously. Briefly, cells were incubated for 3–6minat 37°C with primary antibodies against extracellular epitopes of GABA_A_R γ2 subunit (guinea pig: 1:800/1:1000, rabbit: 1:500, Synaptic Systems) or GABA_A_R α1 subunit (rabbit: 1:500, Synaptic Systems) and washed. Cells were then incubated for 3–5minat 37°C with biotinylated Fab secondary antibodies (goat anti-guinea pig (106-066-003) or goat anti-rabbit (111-067-003),1:500; Jackson Immunoresearch, West Grove, USA) in imaging medium. After washes, cells were incubated for 1 min with streptavidin-coated quantum dots (QDs) (1 nM; Invitrogen, Q10123MP) or anti-rabbit F(ab’)2-QDs emitting at 655 nm (1 nM; Invitrogen, 10592815) in PBS (1X; Invitrogen) in the presence of 10% Casein (v/v, Sigma) to prevent non-specific binding. Washing and incubation steps were all done in imaging medium.

#### Immunocytochemistry

Cells were exposed for 2 h at 37°C to the drugs before labeling. Then, GABA_A_R γ2 or α1 subunits were labeled by incubating living neurons for 20minat 4°C with primary antibodies against extracellular epitopes of GABA_A_R γ2 subunit (guinea pig: 2 μg/mL, Synaptic Systems, 224004; rabbit: 2 μg/mL, Synaptic Systems, 224,003) or GABA_A_R α1 subunit (rabbit:2 μg/mL, Synaptic Systems, 224,203) diluted in the imaging medium. After three washes in imaging medium, neurons were fixed for 15minat room temperature (RT) in paraformaldehyde (PFA, 4% w/v, Sigma) and sucrose (14% w/v, Sigma) solution prepared in PBS (1X). Cells were washed in PBS and permeabilized for 4minat RT with Triton X-100 0.25% (w/v; Invitrogen) in PBS. Then, they were incubated for 30minat RT in normal goat serum (GS, 3% v/v, Invitrogen) in PBS to block non-specific staining. Subsequently, neurons were incubated for 1 h with mouse anti-VGAT antibodies (2 μg/mL, Synaptic Systems, 131,011) in PBS supplemented with GS (3% v/v, Invitrogen). After washes, cells were incubated for 60minat RT with a secondary antibody mix containing FITC-conjugated goat anti mouse (3.75 μg/mL, Jackson Immunoresearch, 115-095-003) and CY3-conjugated goat antirabbit (3.75 μg/mL, Jackson Immunoresearch, 111-095-003) or CY3-conjugated donkey anti guinea pig (3.75 μg/mL, Jackson Immunoresearch, 706-165-148) in PBS-GS blocking solution, washed, and finally mounted on glass slides using Mowiol 4–88 (48 mg/mL, Sigma). Sets of neurons compared for quantification were labeled simultaneously.

#### PALM

Cells were transfected at DIV10 with dendra2-GABA_A_Rγ2^WT^, dendra2-GABA_A_Rγ2L^V262F^ or dendra2-gephyrin constructs. They were then exposed at DIV21 for 2 h at 37°C to the appropriate drugs before fixation in 4% PFA for 15 min and washes in PBS 1X. They were then mounted in a Ludin chamber and imaged in PBS 1X.

#### Calcium imaging

Neurons at DIV21-25 were loaded with 10 μM Fluo-4AM (Life Technologies, 14217) for 5 minat 37°C in imaging medium. After washing excess dye, cells were further incubated for 5–10 min to allow hydrolysis of the AM ester. All incubation steps and washes were performed in imaging medium.

### Quantification and statistical analysis

#### Fluorescence image acquisition and analysis

Image acquisition was performed using a 63× objective (NA 1.32) on a Leica (Nussloch, Germany) DM6000 upright epifluorescence microscope with a 12-bit cooled CCD camera (Micromax, Roper Scientific) run by MetaMorph software (Roper Scientific, Evry, France). Image exposure time was determined on bright cells to obtain best fluorescence to noise ratio and to avoid pixel saturation. All images from a given culture were then acquired with the same exposure time and acquisition parameters. For cluster colocalization analysis, quantification was performed using MetaMorph software (Roper Scientific). For each image, several dendritic regions of interest were manually chosen and a user-defined intensity threshold was applied to select clusters and avoid their coalescence. For quantification of gephyrin or GABA_A_R synaptic clusters, gephyrin or receptor clusters comprising at least 3 pixels and colocalized on at least 1 pixel with VGAT clusters were considered. The number of clusters, the surface area and the integrated fluorescence intensities of clusters were measured. For surface expression analysis, quantification was performed using ImageJ (National Institutes of Health and LOCI, University of Wisconsin). Several dendritic regions of interest were manually chosen and mean average intensity per pixel was measured.

#### Single particle tracking and analysis

Cells were imaged as previously described using an Olympus IX71 inverted microscope equipped with a 60X objective (NA 1.42; Olympus) and an X-Cite 120Q (Excelitas Technologies). Individual images of Gephyrin-FingR-eGFP or clathrin-YFP and QD real time recordings (integration time of 75 ms over 1200 consecutive frames) were acquired with Hamamatsu ImagEM EMCCD camera and MetaView software (Meta Imaging 7.7). Cells were imaged within 50 min following labeling.

QD tracking and trajectory reconstruction were performed with homemade software (MATLAB; The Mathworks, Natick, MA) as described in (Bannai et al., 2006). One to two sub-regions of dendrites were quantified per cell. In cases of QD crossing, the trajectories were discarded from analysis. Trajectories were considered synaptic when overlapping with the synaptic mask of gephyrin clusters, or extrasynaptic for spots two pixels (380 nm) away. Values of the mean square displacement (MSD) plot versus time were calculated for each trajectory by applying the relation:

$$MSD\left(n\tau \right)=\frac{1}{N-n}\sum_{i=1}^{N-n}\left[{\left(x\left(\left(i+n\right)\tau \right)-x\left(i\tau \right)\right)}^{2}+{\left(y\left(\left(i+n\right)\tau \right)-y\left(i\tau \right)\right)}^{2}\right]$$

(Saxton and Jacobson, 1997), where τ is the acquisition time, N is the total number of frames, n and i are positive integers with n determining the time increment. Diffusion coefficients (D) were calculated by fitting the first four points without origin of the MSD versus time curves with the equation: where b is a constant reflecting the spot localization accuracy. The explored area of each trajectory was defined as the MSD value of the trajectory at two different time intervals of at 0.42 and 0.45 s.

#### PALM

PALM imaging was carried out on an inverted N-STORM Nikon Eclipse Ti microscope with a 100x oil-immersion objective (N.A. 1.49) and an Andor iXon Ultra 897 EMCCD camera (image pixel size, 160 nm), using specific lasers for PALM imaging of dendra2 (405 and 561 nm). Movies of ∼20000 frames were acquired at frame rates of 20 ms. The z position was maintained during acquisition by a Nikon Perfect Focus System. Single-molecule localization and 2D image reconstruction was conducted as described in (Specht et al., 2013) by fitting the PSF of spatially separated fluorophores to a 2D Gaussian distribution. PALM images were rendered by superimposing the coordinates of single-molecule detections, which were represented with 2D Gaussian curves of unitary intensity and SDs representing the localization accuracy (σ = 20 nm).In order to correct multiple detections coming from the same Dendra2 molecule (Specht et al., 2013), we identified detections occurring in the vicinity of space (2 x sigma) and time (15 s) as belonging to a same molecule. The surface of GABA_A_R and gephyrin clusters and the densities of molecules per μm^2^ were measured in reconstructed 2D images through cluster segmentation based on detection densities. The threshold to define the border was set to 1000 detections/μm^2^, taking into account the reported gephyrin densities in synapses (Specht et al., 2013). Briefly, all pixels (PALM pixel size = 20 nm) containing <2 detections were considered empty, and their intensity value was set to 0. The intensity of pixels with 2 detections was set to 1. The resulting binary image was analyzed with the function “regionprops” of MATLAB to extract the surface area of each cluster identified by this function. Density was calculated as the total number of detections in the pixels belonging to a given cluster, divided by the area of the cluster. To study SSDs, clusters were then analyzed individually. A threshold was set to 1000 detections/μm ^2^ to define the edges of each SSD and the number, size and density of molecules per SSD were determined.

#### Calcium imaging

Cells were imaged at 37°C in an open chamber mounted on an inverted microscope N-STORM Nikon Eclipse Ti microscope with a 100x oil-immersion objective (N.A. 1.49). Fluo4-AM was illuminated using 472 (± 30) light from a diode. Emitted light was collected using an a 520 (± 35) nm emission emission filter. Time-lapse at 0.033 Hz were acquired with an exposure time of 100 ms for 990 s with an Andor iXon Ultra 897 EMCCD camera (image pixel size, 160 nm) using Nikon software. The analysis was performed on a section of the soma that was in focus at different time points. Fluorescence intensities collected in the soma before (F0) and following (F) bath addition of the drugs, were backgroundsubtracted. The data were analyzed using ImageJ (NIH, USA). Normalization of fluorescence intensity was performed by dividing the mean fluorescence intensity after drug application by the mean fluorescence intensity before drug application.

#### Statistics

For all quantified experiments the experimenters were blind to the condition of the sample analyzed. All experiments were performed at least 3 times from independent cell preparations and transfections, unless stated otherwise in the figure legends. Sampling corresponds to the numberof quantum dots for SPT, the number of cells for ICC, and the number of synapses for PALM. Sample size selection for experiments was based on published experiments, pilot studies as well as in-house expertise. All results were used for analysis except in few cases. Cells with signs of suffering (apparition of blobs, fragmented neurites) were discarded from the analysis. Means are shown ± SEM, median values are indicated with their interquartile range (IQR, 25–75%). Means were compared using the non-parametric Mann-Whitney test (immunocytochemistry, PALM quantifications) using SigmaPlot 12.5 software (Systat Software). Diffusion coefficient and explored area values having non-normal distributions, a non-parametric Kolmogorov-Smirnov test was run underMATLAB (The Mathworks, Natick, MA). For calcium imaging analysis, statistics (paired t-test) were run for each cell on the mean fluorescence intensities calculated before and after drug application (all time points included). Differences were considered significant for pvalues less than 5% (∗p ≤ 0.05; ∗∗p < 0.01; ∗∗∗p < 0.001).

## Data Availability

No standardized datatypes are reported in this paper. All data reported in this paper will be shared by the lead contact upon request. This paper does not report original code. Any additional information required to reanalyze the data reported in this paper is available from the lead contact upon request.

## References

[bib1] Bannai H., Lévi S., Schweizer C., Dahan M., Triller A. (2006). Imaging the lateral diffusion of membrane molecules with quantum dots. Nat. Protoc..

[bib2] Bannai H., Lévi S., Schweizer C., Inoue T., Launey T., Racine V., Sibarita J.B., Mikoshiba K., Triller A. (2009). Activity-dependent tuning of inhibitory neurotransmission based on GABA_A_R diffusion dynamics. Neuron.

[bib3] Bannai H., Niwa F., Sherwood M.W., Shrivastava A.N., Arizono M., Miyamoto A., Sugiura K., Lévi S., Triller A., Mikoshiba K. (2015). Bidirectional control of synaptic GABA_A_R clustering by glutamate and calcium. Cell Rep..

[bib4] Battaglia S., Renner M., Russeau M., Côme E., Tyagarajan S.K., Lévi S. (2018). Activity-dependent inhibitory synapse scaling is determined by gephyrin phosphorylation and subsequent regulation of GABA_A_ receptor diffusion. eNeuro.

[bib5] Brünig I., Scotti E., Sidler C., Fritschy J.M. (2002). Intact sorting, targeting, and clustering of γ-aminobutyric acid A receptor subtypes in hippocampal neurons in vitro. J. Comp. Neurol..

[bib6] Chamma I., Heubl M., Chevy Q., Renner M., Moutkine I., Eugène E., Poncer J.C., Lévi S. (2013). Activity-dependent regulation of the K/Cl transporter KCC2 membrane diffusion, clustering, and function in hippocampal neurons. J. Neurosci..

[bib7] Choquet D., Triller A. (2013). The dynamic synapse. Neuron.

[bib8] Christie S.B., Li R.W., Miralles C.P., Yang B.Y., De Blas A.L. (2006). Clustered and non-clustered GABA_A_ receptors in cultured hippocampal neurons. Mol. Cell. Neurosci..

[bib9] Constals A., Penn A.C., Compans B., Toulmé E., Phillipat A., Marais S., Retailleau N., Hafner A.-S., Coussen F., Hosy E., Choquet D. (2015). Glutamate-induced AMPA receptor desensitization increases their mobility and modulates short-term plasticity through unbinding from Stargazin. Neuron.

[bib10] Crosby K.C., Gookin S.E., Garcia J.D., Hahm K.M., Dell’Acqua M.L., Smith K.R. (2019). Nanoscale subsynaptic domains underlie the organization of the inhibitory synapse. Cell Rep..

[bib11] Dahan M., Lévi S., Luccardini C., Rostaing P., Riveau B., Triller A. (2003). Diffusion dynamics of glycine receptors revealed by single-quantum dot tracking. Science.

[bib12] Dzyubenko E., Rozenberg A., Hermann D.M., Faissner A. (2016). Colocalization of synapse marker proteins evaluated by STED-microscopy reveals patterns of neuronal synapse distribution in vitro. J. Neurosci. Methods.

[bib13] Essrich C., Lorez M., Benson J.A., Fritschy J.M., Lüscher B. (1998). Postsynaptic clustering of major GABA_A_ receptor subtypes requires the γ2 subunit and gephyrin. Nat. Neurosci..

[bib14] Field M., Dorovykh V., Thomas P., Smart T.G. (2021). Physiological role for GABAA receptor desensitization in the induction of long-term potentiation at inhibitory synapses. Nat. Commun..

[bib15] Gielen M., Thomas P., Smart T.G. (2015). The desensitization gate of inhibitory Cys-loop receptors. Nat. Commun..

[bib16] Gielen M., Corringer P.J. (2018). The dual-gate model for pentameric ligand-gated ion channels activation and desensitization. J. Physiol..

[bib17] Gouzer G., Specht C.G., Allain L., Shinoe T., Triller A. (2014). Benzodiazepine-dependent stabilization of GABA_A_ receptors at synapses. Mol. Cell. Neurosci..

[bib18] Gross G.G., Junge J.A., Mora R.J., Kwon H.B., Olson C.A., Takahashi T.T., Liman E.R., Ellis-davies G.C.R., Mcgee A.W., Sabatini B.L. (2013). NeuroResource Recombinant probes for visualizing endogenous synaptic proteins in living neurons. Neuron.

[bib19] Günther U., Benson J., Benke D., Fritschy J.M., Reyes G., Knoflach F., Crestani F., Aguzzi A., Arigoni M., Lang Y. (1995). Benzodiazepine-insensitive mice generated by targeted disruption of the gamma 2 subunit gene of gamma-aminobutyric acid type A receptors. Proc. Natl. Acad. Sci. USA.

[bib20] Haas K.T., Compans B., Letellier M., Bartol T.M., Grillo-Bosch D., Sejnowski T.J., Sainlos M., Choquet D., Thoumine O., Hosy E. (2018). Pre-post synaptic alignment through neuroligin-1 tunes synaptic transmission efficiency. Elife.

[bib21] Hannan S., Minere M., Harris J., Izquierdo P., Thomas P., Tench B., Smart T.G. (2020). GABA_A_R isoform and subunit structural motifs determine synaptic and extrasynaptic receptor localisation. Neuropharmacology.

[bib22] Jacob T.C., Bogdanov Y.D., Magnus C., Saliba R.S., Kittler J.T., Haydon P.G., Moss S.J. (2005). Gephyrin regulates the cell surface dynamics of synaptic GABA_A_ receptors. J. Neurosci..

[bib23] Kralic J.E., Sidler C., Parpan F., Homanics G.E., Morrow A.L., Fritschy J.M. (2006). Compensatory alteration of inhibitory synaptic circuits in cerebellum and thalamus of γ-aminobutyric acid type A receptor α1 subunit knockout mice. J. Comp. Neurol..

[bib24] Kumar A., Basak S., Rao S., Gicheru Y., Mayer M.L., Sansom M.S.P., Chakrapani S. (2020). Mechanisms of activation and desensitization of full-length glycine receptor in lipid nanodiscs. Nat. Commun..

[bib25] Lévi S., Le Roux N., Eugène E., Poncer J.C. (2015). Benzodiazepine ligands rapidly influence GABA_A_ receptor diffusion and clustering at hippocampal inhibitory synapses. Neuropharmacology.

[bib26] Li R.W., Yu W., Christie S., Miralles C.P., Bai J., LoTurco J.J., De Blas A.L. (2005). Disruption of postsynaptic GABA_A_ receptor clusters leads to decreased GABAergic innervation of pyramidal neurons. J. Neurochem..

[bib27] de Luca E., Ravasenga T., Petrini E.M., Polenghi A., Nieus T., Guazzi S., Barberis A. (2017). Inter-synaptic lateral diffusion of GABA_A_ receptors shapes inhibitory synaptic currents. Neuron.

[bib28] Masiulis S., Desai R., Uchański T., Serna Martin I., Laverty D., Karia D., Malinauskas T., Zivanov J., Pardon E., Kotecha A. (2019). GABA_A_ receptor signalling mechanisms revealed by structural pharmacology. Nature.

[bib29] Mortensen M., Ebert B., Wafford K., Smart T.G. (2010). Distinct activities of GABA agonists at synaptic- and extrasynaptic-type GABA_A_ receptors. J. Physiol..

[bib30] Muir J., Arancibia-Carcamo I.L., MacAskill A.F., Smith K.R., Griffin L.D., Kittler J.T. (2010). NMDA receptors regulate GABA_A_ receptor lateral mobility and clustering at inhibitory synapses through serine 327 on the γ2 subunit. Proc. Natl. Acad. Sci. USA.

[bib31] Mukherjee J., Kretschmannova K., Gouzer G., Maric H.-M., Ramsden S., Tretter V., Harvey K., Davies P.a., Triller a., Schindelin H., Moss S.J. (2011). The residence time of GABA_A_Rs at inhibitory synapses is determined by direct binding of the receptor α1 subunit to gephyrin. J. Neurosci..

[bib32] Overstreet L.S., Jones M.V., Westbrook G.L. (2000). Slow desensitization regulates the availability of synaptic GABA(_A_) receptors. J. Neurosci..

[bib33] Pennacchietti F., Vascon S., Nieus T., Rosillo C., Das S., Tyagarajan S.K., Diaspro A., Del Bue A., Petrini E.M., Barberis A., Cella Zanacchi F. (2017). Nanoscale molecular reorganization of the inhibitory postsynaptic density is a determinant of GABAergic synaptic potentiation. J. Neurosci..

[bib34] Petrini E.M., Barberis A. (2014). Diffusion dynamics of synaptic molecules during inhibitory postsynaptic plasticity. Front. Cell. Neurosci..

[bib35] Petrini E.M., Lu J., Cognet L., Lounis B., Ehlers M.D., Choquet D. (2009). Endocytic trafficking and recycling maintain a pool of mobile surface AMPA receptors required for synaptic potentiation. Neuron.

[bib36] Petrini E.M., Nieus T., Ravasenga T., Succol F., Guazzi S., Benfenati F., Barberis A. (2011). Influence of GABA_A_R monoliganded states on GABAergic responses. J. Neurosci..

[bib37] Petrini E.M., Ravasenga T., Hausrat T.J., Iurilli G., Olcese U., Racine V., Sibarita J.-B., Jacob T.C., Moss S.J., Benfenati F. (2014). Synaptic recruitment of gephyrin regulates surface GABA_A_ receptor dynamics for the expression of inhibitory LTP. Nat. Commun..

[bib38] Renner M., Schweizer C., Bannai H., Triller A., Lévi S. (2012). Diffusion barriers constrain receptors at synapses. PLoS One.

[bib39] Rust M.J., Lakadamyali M., Zhang F., Zhuang X. (2004). Assembly of endocytic machinery around individual influenza viruses during viral entry. Nat. Struct. Mol. Biol..

[bib40] Saliba R.S., Kretschmannova K., Moss S.J. (2012). Activity-dependent phosphorylation of GABA_A_ receptors regulates receptor insertion and tonic current. EMBO J..

[bib41] Saxton M.J., Jacobson K. (1997). Single-particle tracking: applications to membrane dynamics. Annu. Rev. Biophys. Biomol. Struct..

[bib42] Schweizer C., Balsiger S., Bluethmann H., Mansuy I.M., Fritschy J.M., Mohler H., Lüscher B. (2003). The γ2 subunit of GABA_A_ receptors is required for maintenance of receptors at mature synapses. Mol. Cell. Neurosci..

[bib43] Smith K.R., Muir J., Rao Y., Browarski M., Gruenig M.C., Sheehan D.F., Haucke V., Kittler J.T. (2012). Stabilization of GABA(A) receptors at endocytic zones is mediated by an AP2 binding motif within the GABA(A) receptor β3 subunit. J. Neurosci..

[bib44] Specht C.G. (2019). Fractional occupancy of synaptic binding sites and the molecular plasticity of inhibitory synapses. Neuropharmacology.

[bib45] Specht C.G., Izeddin I., Rodriguez P., ElBeheiry M., Rostaing P., Darzacq X., Dahan M., Triller A. (2013). Quantitative nanoscopy of inhibitory synapses: counting gephyrin molecules and receptor binding sites. Neuropharmacology.

[bib46] Studer R., Von Boehmer L., Haenggi T., Schweizer C., Benke D., Rudolph U., Fritschy J.M. (2006). Alteration of GABAergic synapses and gephyrin clusters in the thalamic reticular nucleus of GABA_A_ receptor α3 subunit-null mice. Eur. J. Neurosci..

[bib47] Triller A., Choquet D. (2008). New concepts in synaptic biology derived from single-molecule imaging. Neuron.

[bib48] Triller A., Renner M., Choquet D. (2020). Dynamics and organization of proteins in the neuronal plasma membrane. Neuropharmacology.

[bib49] Yang X., Specht C.G. (2019). Subsynaptic domains in super-resolution microscopy: the treachery of images. Front. Mol. Neurosci..

