# Supplementary material for: Conformational state-dependent regulation of GABAA receptor diffusion and subsynaptic domains
Source: iScience. 2022 Oct 29;25(11):105467. doi: 10.1016/j.isci.2022.105467 (PMC9663900; doi:10.1016/j.isci.2022.105467)
Supplement: Document S1. Figures S1–S7 [file mmc1.pdf]

iScience, Volume *25*

Supplemental information

Conformational state-dependent regulation of $GABA_A$ receptor diffusion and subsynaptic domains

Zaha Merlaud, Xavier Marques, Marion Russeau, Ursula Saade, Maelys Tostain, Imane Moutkine, Marc Gielen, Pierre-Jean Corringer, and Sabine Lévi

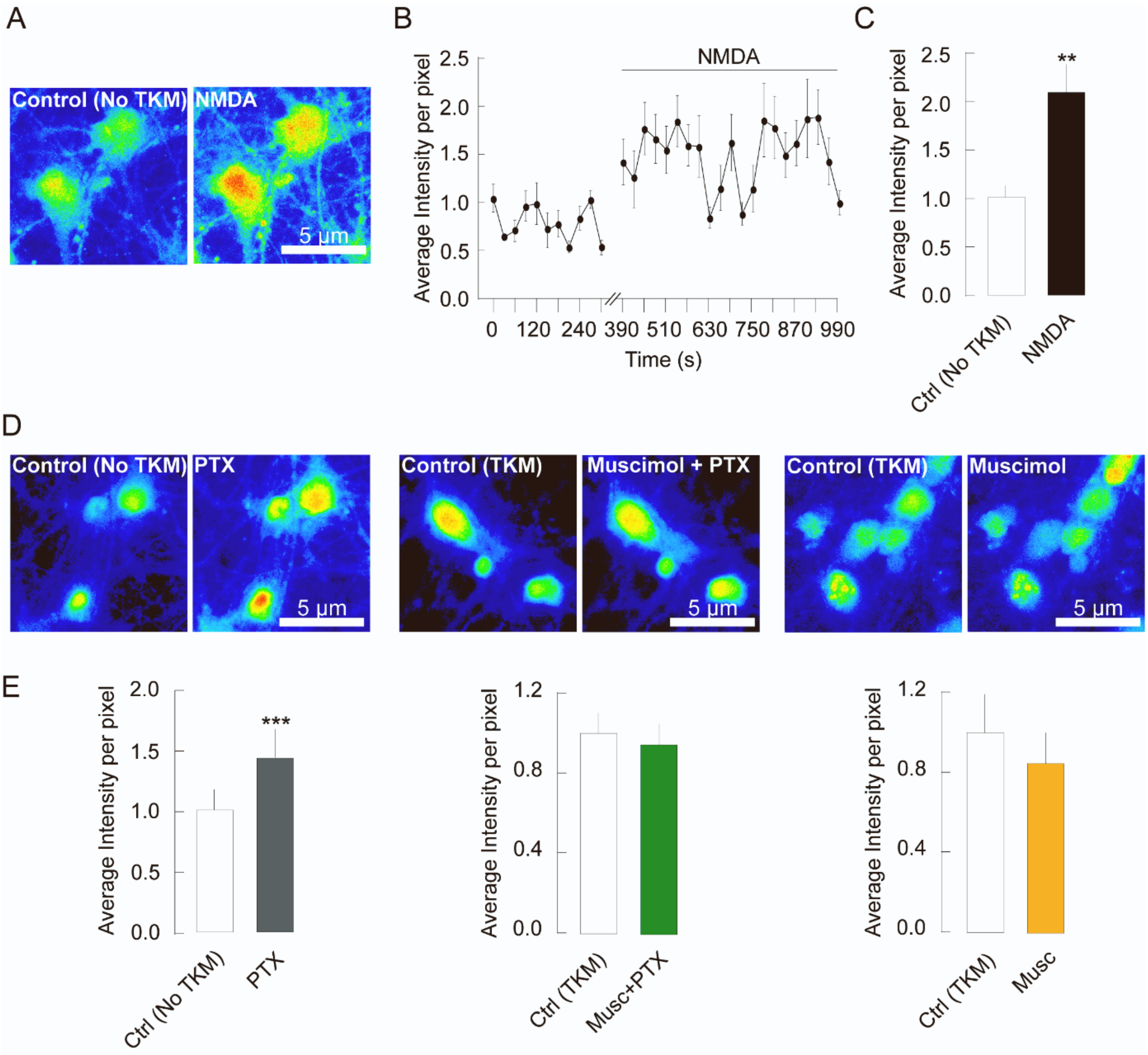

Figure S1. Experimental conditions favoring the active and desensitized conformational states of the $GABA_AR$ have no impact on neuronal ion flow. A, Pseudocolor images of neurons loaded with Fluo4-AM, before (Control, No TKM) and after (NMDA, 50 µM) exposure. Warmer colors correspond to higher Fluo4-AM fluorescence intensities. Scale bar, 5 µm. B, Calcium levels measured in neuronal somata shown as average intensity per pixel as a function of time before and following (black bar) NMDA application. C, Mean intensity per pixel of Fluo4-AM in neuronal somata before (white) and after (black) NMDA application. Note the increase in intracellular calcium level upon NMDA treatment. n= 10 cells, p= 0.002. D, Pseudocolor images of neurons loaded with Fluo4-AM, before (Control, No TKM) and after PTX treatment in absence of TKM, or before (Control) and after muscimol + PTX, or muscimol treatment in presence of TKM, as indicated. Warmer colors correspond to higher Fluo4-AM fluorescence intensities. Scale bar, 5 µm. E, Average Fluo4-AM fluorescence intensity per pixel before (white) application of the drug and after PTX (grey), muscimol + PTX (green) or muscimol (orange) treatment. Note the increase in intracellular calcium level upon PTX treatment in absence of TKM but no change in intra-neuronal calcium level upon muscimol or muscimol + PTX exposure in presence of TKM. PTX, n= 20 cells, p= 0.0004; Musc + PTX, n= 22 cells, p= 0.3053; Musc, n= 19 cells, p= 0.0602. Data are presented as mean ± SEM. In all graphs, **, p<1.0 10^{-2}, ***, p<1.0 10^{-3} (Wilcoxon matched-pairs signed rank test). Related to Star Methods.

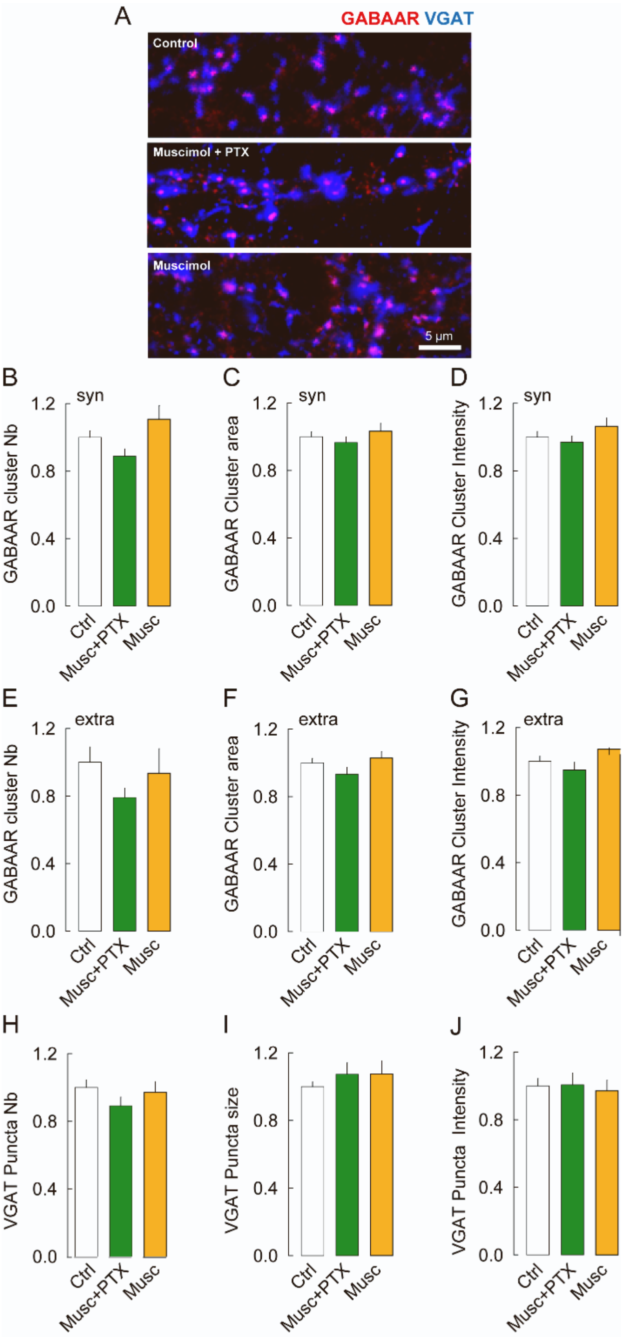

Figure S2. Conventional fluorescence microscopy does not reveal any changes in GABA$_A$Rγ2 and VGAT staining upon GABA$_A$R conformational changes. A, Representative images of primary hippocampal neurons stained for GABA$_A$Rγ2 (red), and VGAT (blue) and imaged with conventional fluorescence microscopy. Neurons were exposed to muscimol + PTX or muscimol alone to promote the active or desensitized conformational states of the GABA$_A$R and compared with mock treated control. Scale bar, 5 µm. B-G, Quantification of GABA$_A$Rγ2 cluster number (B, E), area (C, F), and intensity (D, G) at synapses (B-D) and extrasynaptic sites (E-G) shows that favoring the GABA$_A$R active or desensitized conformations does not change the size and intensity of GABA$_A$Rγ2 clusters at synapses and at extrasynaptic sites. Ctr, n= 78 cells, Musc + PTX, n= 67 cells, Musc, n= 37 cells, 3-4 cultures. Synaptic: Musc + PTX, Cluster Nb p=0.09, area p=0.5, intensity p=0.6; Musc, Cluster Nb p=0.4, area p=0.7, intensity p=0.6; Extrasynaptic: Musc + PTX, Cluster Nb p=0.1, area p=5.0 10^{-3}; intensity p=0.1; Musc, Cluster Nb p=0.1, area p=0.3, intensity p=0.1. H-J, Quantification of VGAT cluster number (H), area (I), and intensity (J) shows no impact of favoring the active or desensitized conformational states on presynaptic terminals. Ctr, n= 78 cells, Musc + PTX, n= 47 cells, Cluster Nb p=0.2, area p=0.4, intensity p=0.7, Musc, n= 37 cells, Cluster Nb p=0.3, area p=0.6, intensity p=0.6, 3 cultures. Data shown as mean ± SEM. Values were normalized to the corresponding control values. (Mann–Whitney rank sum test). Related to Figure 4.

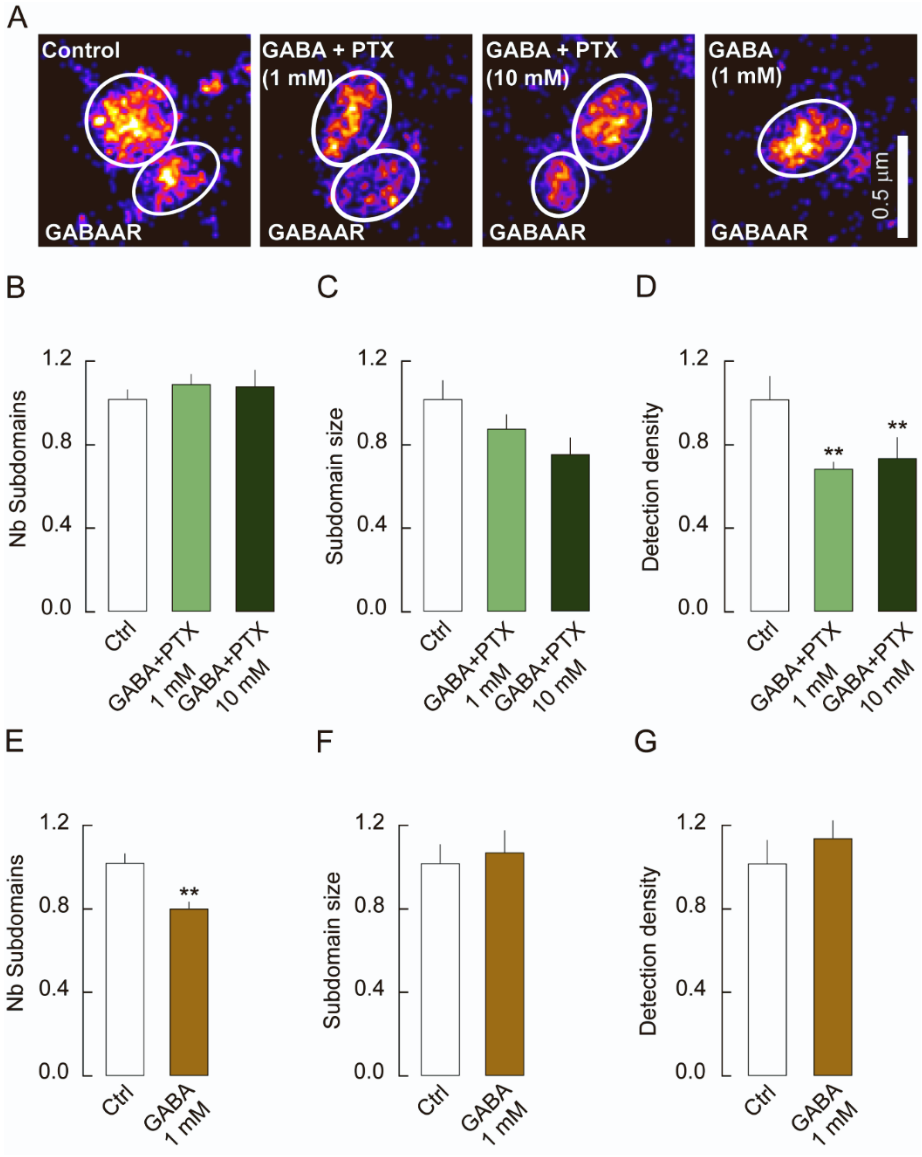

Figure S3. GABA agonist affects the conformation of the receptor and its synaptic subdomain organization. A, Representative images of dendra2-GABA$_A$Rγ2 in control condition or in the presence of GABA + PTX (GABA, 1 or 10 mM) or GABA (1 mM) alone to favor the active and desensitized conformational states, respectively. Scale bar, 0.5 µm. Note the reduced labeling of the SSDs in the GABA + PTX condition whatever the concentration of GABA used (1 or 10 mM) and the reduced number of SSDs in the GABA condition, as compared to the control condition. B-D, Quantification of the number (B) of dendra2-GABA$_A$Rγ2 SSDs per synapse, the size of SSDs (C) and the density of molecules detected per SSD (D) upon GABA + PTX (GABA 1 mM) and GABA + PTX (GABA 10 mM) treatment. The density of SSDs and their size were not affected by these treatments while a significant decrease of the density of single molecules detected per SSD was observed upon GABA + PTX treatment, regardless of GABA concentration. B, Ctrl, n= 116 synapses, GABA + PTX (GABA 1 mM), n= 160 synapses, p= 0.5426, GABA + PTX (GABA 10 mM), n= 67 synapses, p= 0.8732. C-D, Ctrl, n= 171 subdomains, GABA + PTX (10mM), n= 106 subdomains, C, p= 0.1824, D, p= 0.0037, GABA + PTX (1 mM), n= 252 subdomains, C, p= 0.3761, D, p= 0.0013, 2 cultures. E-G, Quantification of the number (E) of dendra2-GABA$_A$Rγ2 subdomains per synapse, the size of SSDs (F) and the density of molecules detected per SSD (G) upon GABA (1 mM) treatment. The density of SSDs was decreased upon GABA treatment whereas the size and the density of single molecules detected per SSD remained unchanged. E, Ctrl, n= 116 synapses, GABA, n= 75 synapses, p= 0.0033, 2 cultures. E-F, Ctrl, n= 171 subdomains, GABA, n= 87 subdomains, E, p= 0.0747, F, p= 0.8165, 2 cultures. Data are presented as mean ± SEM. **, $p<1.0\ 10^{-2}$ (Mann–Whitney rank sum test). Related to Figure 4.

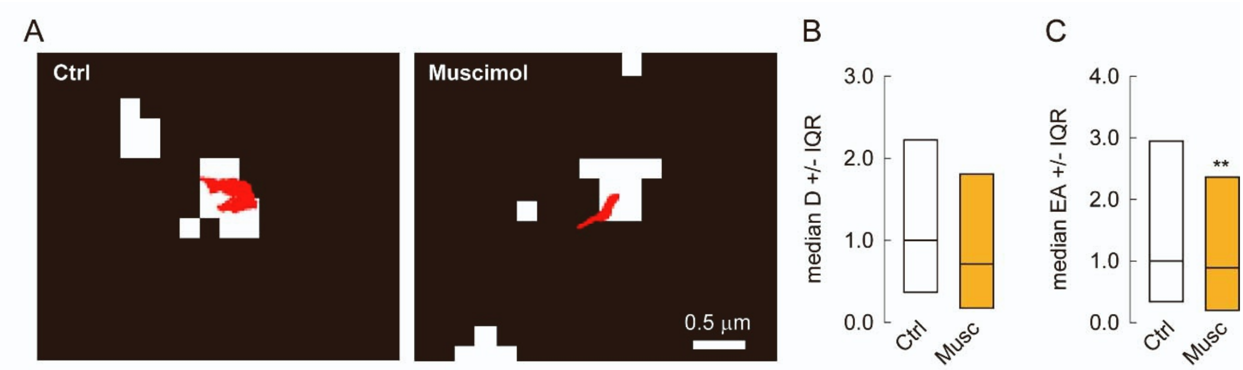

Figure S4. Increased confinement of the desensitized GABA$_A$Rγ2 within endocytic zones. A, Trajectories of GABA$_A$Rγ2 (red) overlaid with fluorescent clusters of YFP-clathrin (white) to identify trajectories in endocytic zones. Scale bar, 0.5 µm. Note the reduced surface exploration of GABA$_A$Rγ2 in the desensitized conformation (induced with muscimol) inside endocytic regions compared to the control condition. B-C, Diffusion coefficients (B) and explored area (C) of GABA$_A$Rγ2 inside endocytic zones in control (white) *vs* muscimol (orange) conditions. B, Ctrl, n= 303 QDs, Musc, n= 156 QDs, p=7.3 10^{-2}; C, Ctrl, n= 906 QDs, Musc, n= 468 QDs, p=1.0 10^{-3}. In B-C, data are presented as median values ± 25%–75% IQR from 3 independent cultures. Values were normalized to the corresponding control values. **, p<1.0 10^{-2} (Kolmogorov–Smirnov test). Related to Figure 7.

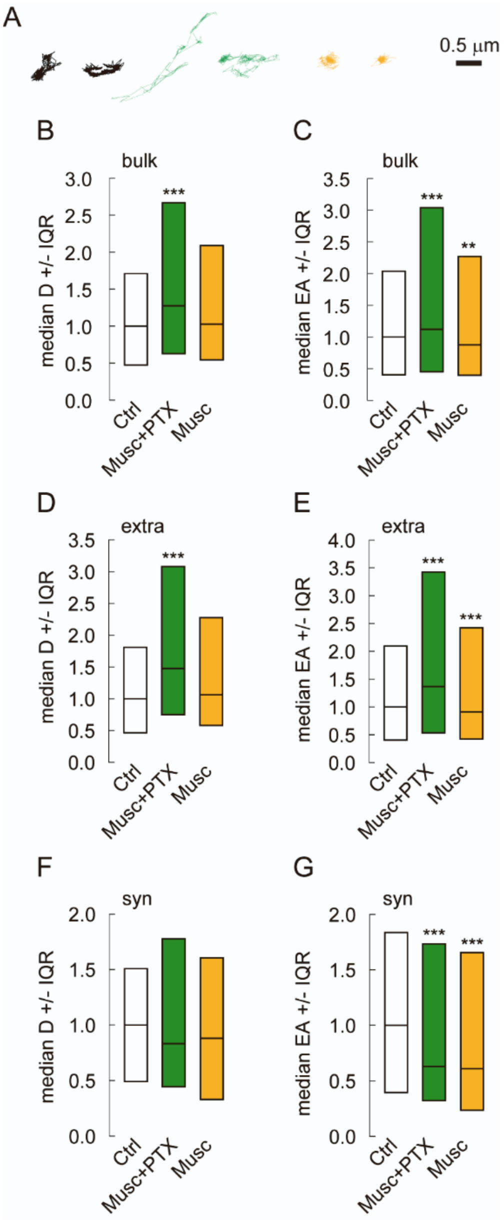

Figure S5. Influence of the $GABA_AR$ conformational states on the membrane dynamics of the α1 subunit. A, Trajectories of $GABA_AR$ α1 subunit (for bulk population of QDs) in the active (green) and desensitized (orange) conformational states favored with muscimol + PTX or muscimol treatment as compared to the control condition (white). Scale bar, 0.5 µm. $GABA_AR$α1 displayed increased surface exploration in the active conformation and reduced exploration in the desensitized states. B-C, Diffusion coefficients (B) and explored area (C) (for bulk population of QDs) of $GABA_AR$α1 in muscimol + PTX (green) or muscimol (orange) conditions. B, Ctrl, n= 538 QDs, Musc + PTX, n= 647 QDs, p=1.0 10^{-6}, Musc, n= 729 QDs, p=0.08, 3 cultures; C, Ctrl, n= 1599 QDs, Musc + PTX, n= 1931 QDs, p=2.7 10^{-9}, Musc, n= 2156 QDs, p=4.6 10^{-4}, 3 cultures. D-E, Diffusion coefficients (D) and explored area (E) (for extrasynaptic population of QDs) of $GABA_AR$α1 in muscimol + PTX (green) or muscimol (orange) conditions. D, Ctrl, n= 349 QDs, Musc + PTX, n= 493 QDs, p=4.7 10^{-7}, Musc, n= 616 QDs, p=9.1 10^{-2}, 3 cultures; E, Ctrl, n= 1161 QDs, Musc + PTX, n= 1473 QDs, p=2.2 10^{-12}, Musc, n= 1842 QDs, p=6.6 10^{-2}, 3 cultures. F-G, Diffusion coefficients (F) and explored area (G) (for synaptic population of QDs) of $GABA_AR$α1 in conditions favoring the active (green) or desensitized (DS, orange) conformational states. F, Ctrl, n= 149 QDs, Musc + PTX, n= 154 QDs, p=0.12, Musc, n= 113 QDs, p=6.5 10^{-2}, 3 cultures; G, Ctrl, n= 441 QDs, Musc + PTX, n= 459 QDs, p=5.1 10^{-6}, Musc, n= 333 QDs, p=3.7 10^{-6}, 3 cultures. In B-G, data are presented as median values ± 25%–75% IQR. In all graphs, values were normalized to the corresponding control values. **, p<0.01; ***, p<0.001 (Kolmogorov–Smirnov test). Related to Figure 1 and Figure S6.

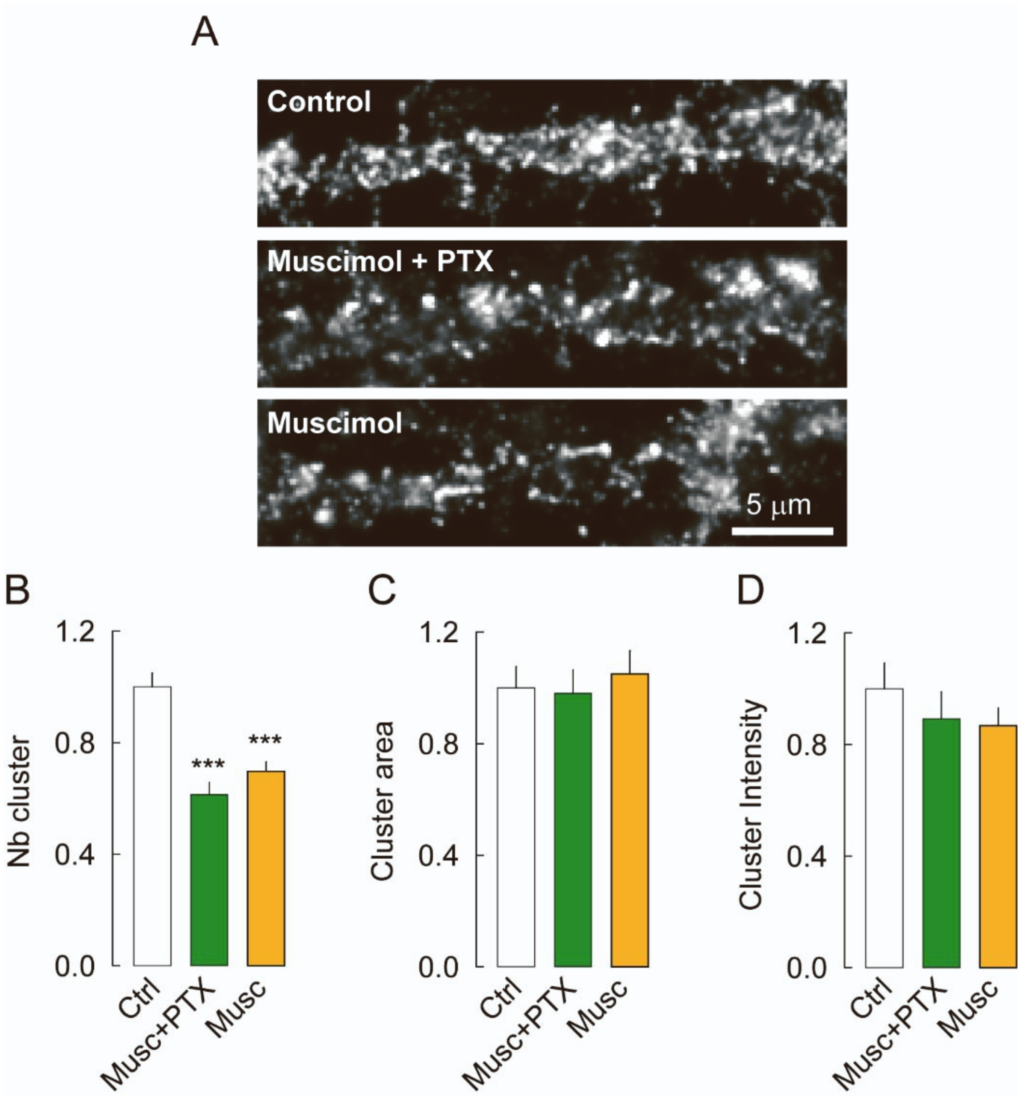

Figure S6. GABA$_A$R conformational changes affect the clustering of the α1 subunit at synapses. A, Representative images of primary hippocampal neurons stained for GABA$_A$Rα1 and imaged with conventional fluorescence microscopy. Neurons were exposed to drugs (muscimol + PTX or muscimol) favoring the active or desensitized conformational states and compared with mock treated control. Scale bar, 5 µm. B-D, Quantification of GABA$_A$Rα1 cluster number (B), area (C), and intensity (D) at synapses shows that favoring the GABA$_A$R active (green) or desensitized (orange) conformational states reduce the density of GABA$_A$Rα1 clusters at synapses. Ctrl, n= 57 cells, Musc + PTX, n= 45 cells, Musc, n= 56 cells, 3-4 cultures. Musc + PTX, Cluster Nb $p<10^{-3}$, area $p=4.2\ 10^{-2}$, intensity $p=9.3\ 10^{-2}$; Musc, Cluster Nb $p<10^{-3}$, area p=0.8, intensity p=0.5. Data shown as mean ± SEM. Values were normalized to the corresponding control values. ***, p<0.001 (Mann–Whitney rank sum test). Related to Figure 4, Figure S5 and Figure S7.

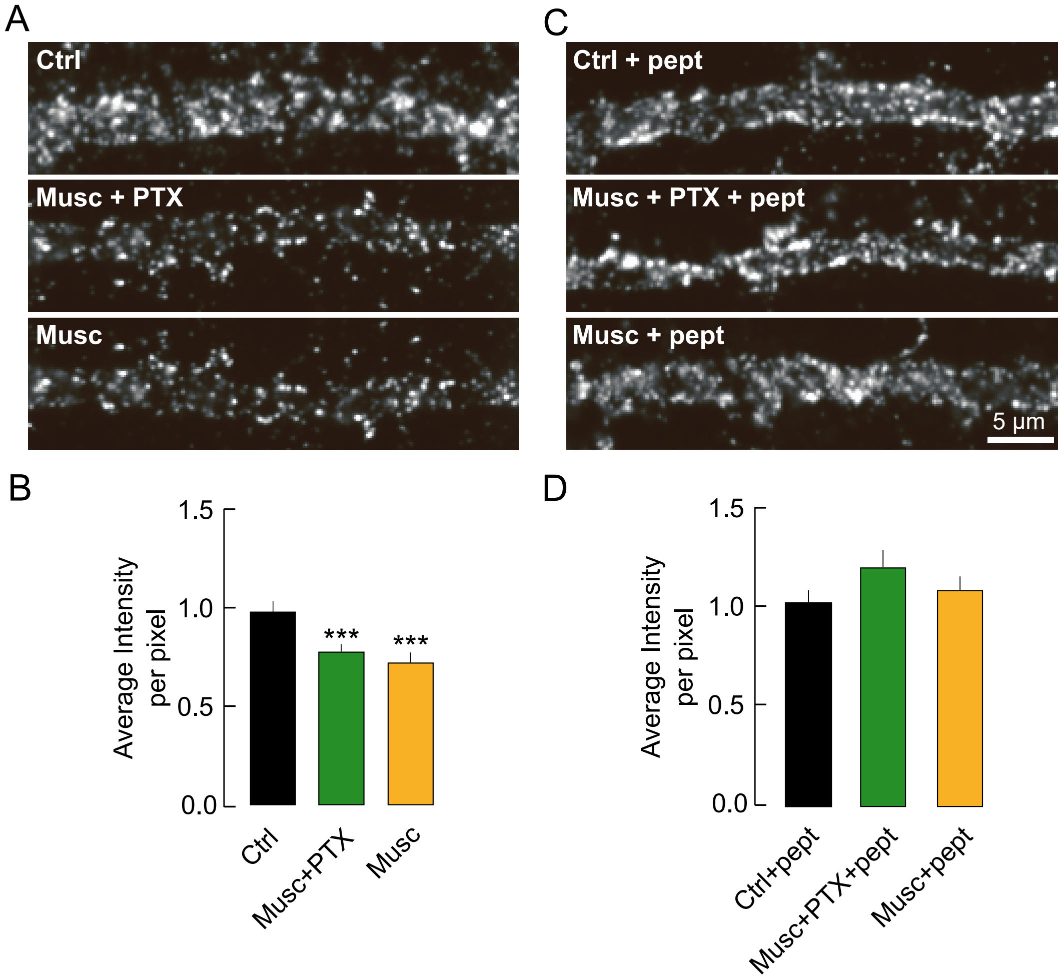

Figure S7. Promoting the active and desensitized conformation of the GABA$_A$R induces endocytosis of the α1 subunit. A, Representative images of hippocampal cultured neurons stained for α1 GABA$_A$R with conventional epifluorescence. Neurons exposed to drugs (Muscimol + PTX or Muscimol) to promote the active or desensitized conformational states, were stained live for the α1 subunit to label the neuronal surface only, and compared with control cells. Scale bar, 5 µm. B, Quantification of the average intensity per pixel of surface α1 subunit shows that promoting the open or desensitized conformation decreases α1 surface expression. Ctrl, n= 49 cells, Musc + PTX, n= 49 cells, p= 0.0005, Musc, n= 48 cells, p= 8.5 10^{-5}, 3 cultures. C, Representative images of surface α1 staining in neurons exposed to drugs (muscimol + PTX or muscimol vs control) in presence of endocytosis inhibitory peptide. Scale bar, 5 µm. D, Quantification of the average intensity per pixel of surface α1 GABA$_A$R shows no loss of α1 surface expression in muscimol or muscimol + PTX conditions vs. control upon blockade of clathrin-mediated endocytosis. Ctrl + pept, n= 9 cells, Musc + PTX + pept, n= 9 cells, p= 0.1135, Musc + pept, n= 9 cells, p= 0.4220, 1 culture. Data shown as mean ± SEM. ***, p<1.0 10^{-3} (Mann-Whitney rank sum test). Related to Figure 7, Figure S4, S5 and S6.
